# β-carboline chemical signals induce reveromycin production through a LuxR family regulator in *Streptomyces* sp. SN-593

**DOI:** 10.1038/s41598-020-66974-y

**Published:** 2020-06-23

**Authors:** Suresh Panthee, Naoko Kito, Teruo Hayashi, Takeshi Shimizu, Jun Ishikawa, Hiroshi Hamamoto, Hiroyuki Osada, Shunji Takahashi

**Affiliations:** 1 RIKEN Center for Sustainable Resource Science, Natural Product Biosynthesis Research Unit, Wako, Hirosawa 2-1, 351-0198 Saitama, Japan; 2 Teikyo University Institute of Medical Mycology, Otsuka 359, Hachioji, Tokyo Japan; 3 RIKEN Center for Sustainable Resource Science, Chemical Biology Research Group, Wako, Hirosawa 2-1, 351-0198 Saitama, Japan; 4 Department of Bioactive Molecules, National Institute of Infectious Diseases, Toyama 1-23-1, Shinjuku, Tokyo 162-8640 Japan

**Keywords:** Chemical ecology, Mechanism of action, Small molecules, Applied microbiology, Soil microbiology

## Abstract

*Actinomycetes* bacteria produce diverse bioactive molecules that are useful as drug seeds. To improve their yield, researchers often optimize the fermentation medium. However, exactly how the extracellular chemicals present in the medium activate secondary metabolite gene clusters remains unresolved. BR-1, a β-carboline compound, was recently identified as a chemical signal that enhanced reveromycin A production in *Streptomyces* sp. SN-593. Here we show that BR-1 specifically bound to the transcriptional regulator protein RevU in the reveromycin A biosynthetic gene cluster, and enhanced RevU binding to its promoter. RevU belongs to the LuxR family regulator that is widely found in bacteria. Interestingly, BR-1 and its derivatives also enhanced the production of secondary metabolites in other *Streptomyces* species. Although LuxR-*N*-acyl homoserine lactone systems have been characterized in Gram-negative bacteria, we revealed LuxR-β-carboline system in *Streptomyces* sp. SN-593 for the production of secondary metabolites. This study might aid in understanding hidden chemical communication by β-carbolines.

## Introduction

Microorganisms utilize a variety of communication systems that enable them to send and receive chemical signals to one another^1^. Quorum sensing is one cell-cell communication mechanism. Gram-negative bacteria produce hormone-like signals, such as the release of acyl-homoserine lactones, to control cellular responses^2,3^. *Streptomyces*, a soil-dwelling gram-positive bacteria, utilize autoregulatory hormones to produce a variety of secondary metabolites (SMs)^4^. The classical example of such hormone is A-factor in *Streptomyces griseus*^5^, and the structurally diverse autoregulators such as 2-alkyl-4-hydroxymethylfuran-3-carboxylic acids in *Streptomyces coelicolor*^6^, PI factor in *Streptomyces natalensis*^7^, avenolide in *Streptomyces avermitilis*^8^, VB-A in *Streptomyces virginiae*^9^, IM-2 in *Streptomyces* sp. FRI-5^10^, SCB1 in *Streptomyces coelicolor*^11^, and SRB1 in *Streptomyces rochei*^12^ are also known to promote morphological development and regulating secondary metabolism. These have been extensively reviewed elsewhere^13–15^. On the other hand, chemical signals derived from extra-species/environmental stimuli such as hormaomycin^16^, goadsporin^17^, promomycin^18^, antibiotic-remodeling compounds (ARCs)^19^, and rare earth elements^20^ also induce morphogenesis and SM production in *Streptomyces* species at higher concentration range compared to autoregulators. Co-culturing *Streptomyces* with mycolic acid-containing bacteria was found to also induce SM production^21^. Except for some ARCs, which inhibit fatty acid biosynthesis, the mechanisms of how extracellular chemical signals activate SM biosynthesis have not been clarified.

**Figure 1:**
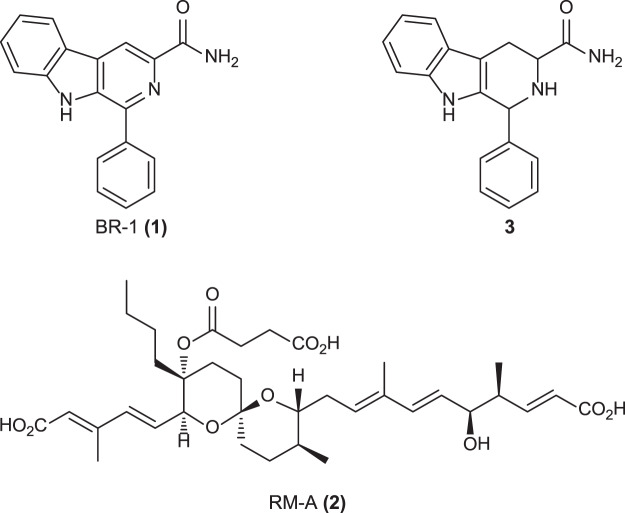
Chemical structures of BR-1 (**1**), RM-A (**2**), and tetrahydro form of BR-1 (**3**).

Reveromycin (RM) A was identified from *Streptomyces* sp. SN-593 as an inhibitor of the mitogenic activity of epidermal growth factor^22^. It also inhibits bone resorption specifically in osteoclast^23^. The RM biosynthetic gene cluster consists of 21 genes, including three transcriptional regulators^24^. Among the regulators, RevU belongs to the LuxR family regulators that harbour a Walker A and B motifs at the N-terminus and a DNA-binding helix-turn-helix domain at the C-terminus^25^. We found that RM production was triggered by the addition of tomato juice to the culture medium, and uncovered the biosynthetic gene cluster for RM-A production in *Streptomyces* sp. SN-593^24^. This led us to speculate that extracellular chemical signals present in nature can enhance SM production. Such chemicals should facilitate the isolation of novel natural products without genetic engineering. However, all attempts to purify chemical signals from tomato did not succeed due to its low-level presence. As an alternative approach, we screened small molecules from the RIKEN Natural Products Depository (NPDepo) consisting of natural products, natural product derivatives, and synthetic chemicals^26,27^. Then, we successfully identified a β-carboline lead compound that enhanced RM production. Based on the structure-activity relationship study, we succeeded to create BR-1 (**1**), which induced RM-A (**2**) production at as little as 0.35 μM concentration in *Streptomyces* sp. SN-593 (Fig. 1)^28^. BR-1 enhanced RM production without affecting cell growth and was demonstrated as an external chemical trigger for increasing SM^28^. In this study, we define BR-1 as biomediator to distinguish them from autoregulators, which are produced intracellularly to regulate functions in the cells that produced them. Based on a chemical biology approach, we identified that the target of β-carboline was a LuxR family transcriptional regulator in the RM gene cluster. Production of autoinducer and subsequent cell responses through LuxR regulators are well characterized in Gram-negative bacteria^29,30^. Here, we discovered that the hidden chemical signal by β-carboline alkaloids through LuxR family regulator was linked with the production of RMs in *Streptomyces* sp. SN-593.

## Results

### Enhancement of RM production by BR-1 requires LuxR family regulator RevU

**Figure 2:**
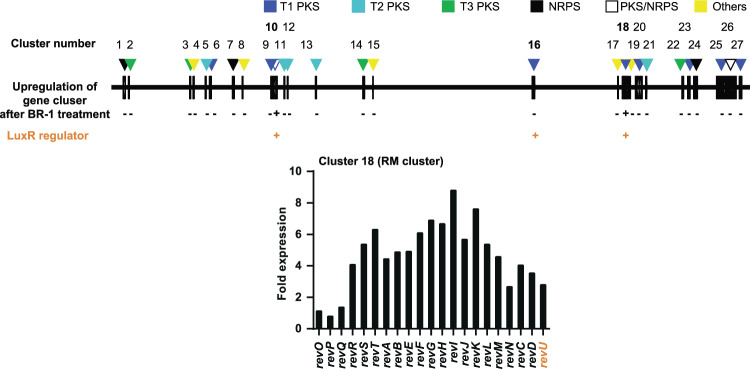
Effects of BR-1 on *Streptomyces* sp. SN-593. Linear map of the **2** producer *Streptomyces* sp. SN-593 genome with putative 27 gene clusters. Following RNA-sequence analysis, fold-expressions of all gene clusters were analysed (Table S1). Gene-cluster upregulation by BR-1 is indicated by ‘+’ and ‘−’. The presence of LuxR family transcriptional regulator in the cluster is indicated by ‘+’. T1PKS: type I PKS; T2PKS: type II PKS; T3PKS: type III PKS; NRPS: nonribosomal peptide synthetase.

**Figure 3:**
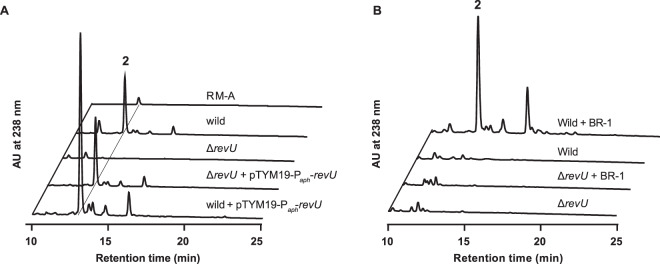
Involvement of RevU in RM biosynthesis. (**A**) After culture with RM high producing medium (RM-PM)^24^, metabolite profiles of wild-type, Δ*revU* mutant, and wild-type and Δ*revU* mutant transformed with the *revU* gene controlled by the *aphII* promoter were analysed. (**B**) After culture with RM low producing medium (SY-B) in the presence or absence of BR-1, metabolic profiles of wild-type and Δ*revU* mutant were analysed.

To understand how BR-1 (1) (Fig. 1) signal upregulated the RM-A gene cluster, we first analysed the genome of *Streptomyces* sp. SN-593 and annotated its gene functions (Table S1). Then we performed RNA-sequence analysis in the presence and absence of BR-1 (Fig. 2). Although we did not observe metabolites, we found that cluster 10, encoding a type I polyketide synthase (PKS), was moderately upregulated after BR-1 treatment (Table S1). mRNA expression of most genes in cluster 18 (RM-A gene cluster) was significantly increased in the presence of BR-1, compared to untreated cells (Fig. 2). Among the 3 regulatory genes, *revP, revQ*, and *revU*, the fold-expression values compared to BR-1 untreated cells were 0.8, 1.4, and 2.8 (Table S1). Real-time PCR was also performed to confirm the enhanced expression of the *revU* gene after BR-1 treatment (Fig. S1A,B). Moreover, *revU* gene disruption (Fig. S2A,B) resulted in the downregulation of genes involved in RM-A biosynthesis (Fig. S2C,D). We also analysed RM production in the *revU* gene disruptant. The Δ*revU* mutant showed completely abolished RM production, which was recovered by re-introducing the *revU* gene under the control of the *aphII* promoter (Fig. 3A). Introducing the *revU* gene in the wild-type strain led to enhanced production of RMs (Fig. 3A). Next, we examined whether BR-1 treatment could recover the loss of RM production caused by disrupting the *revU* gene. We found BR-1 treatment did not recover RM production (Fig. 3B), suggesting the importance of RevU in BR-1-mediated RM production. Taken together, these data suggested that RevU might function as a key regulatory molecule linked with the biomediator activity of BR-1.

### RevU as the molecular target of BR-1

**Figure 4:**
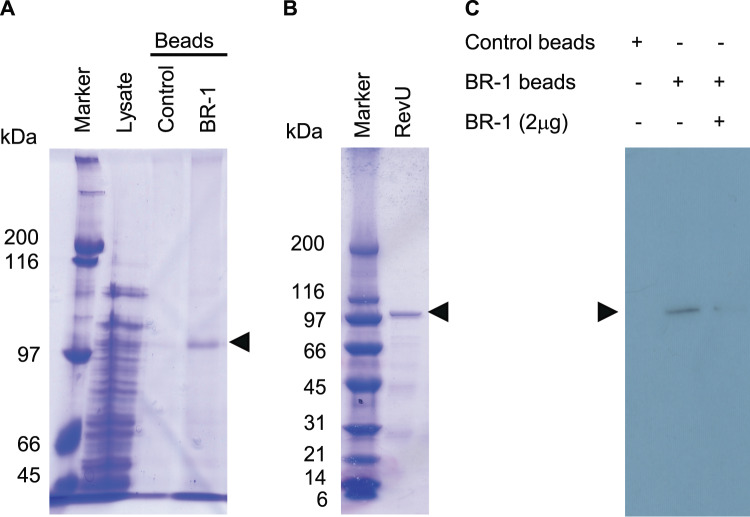
Analysis of BR-1-binding proteins. (**A**) Control and BR-1 beads were mixed with cell lysate (2 mg of protein) from *S. lividans* TK23 expressing RevU and the bound proteins were analysed by 7.5% SDS-PAGE. Lane 1, protein marker. Lane 2, cell lysate. Lanes 3 and 4, cell lysates incubated with control and BR-1 beads, respectively. (**B**) SDS-PAGE analysis of the purified His6-tagged RevU. (**C**) Competition of RevU binding to BR-1 beads using free BR-1. See Fig. S3 for original uncropped images.

To identify the molecular target of BR-1, we prepared BR-1-conjugated agarose beads using the photoaffinity crosslinking method^31,32^. The beads were mixed with the cell lysate of *Streptomyces* sp. SN-593. However, we failed to detect a binding protein because of the low abundance of target protein in the cell lysate. To examine specific interactions between BR-1 and candidate proteins, we expressed RevU protein in *Streptomyces lividans* strain TK23, and the cell lysate was incubated with the BR-1-conjugated beads. Even in the presence of various proteins in crude extracts, we successfully identified a single protein that bound to the BR-1 beads (Figs. 4A and S3). Matrix-assisted laser desorption/ionization-time of flight mass spectrometry (MALDI-TOF/MS) analysis clearly demonstrated that the BR-1-binding protein was RevU (Table S2). Next, we obtained highly purified RevU using the synthetic *revU* gene optimized for heterologous expression in *E. coli* (Fig. 4B and Table S3), because RevU expression in *S. lividans* TK23 was low to obtain a highly purified preparation with high yields. To evaluate the interaction between RevU and BR-1, we tested the competition between BR-1 beads and free BR-1 for RevU binding. We confirmed that His6-tagged RevU bound to BR-1 beads in the absence of free BR-1, but not in the presence of free BR-1 (Fig. 4C), implicating RevU as a molecular target of BR-1. To further characterize the RevU–BR-1 interaction, we conducted surface plasmon resonance (SPR) analysis and observed a quick association and dissociation of BR-1 (Fig. S4A). Interestingly, the equilibrium affinity constant of 1.31 μM calculated for BR-1 was almost identical to its bioactive concentration (Fig. S4B)^28^. Additionally, we have found that the tetrahydro form of BR-1 (**3**) (Fig. 1) did not induce the production of RMs^28^. As expected, the sensorgram for **3** did not reach saturation at a concentration of up to 87 μM, indicative of non-specific binding (Fig. S4C,D).

**Figure 5:**
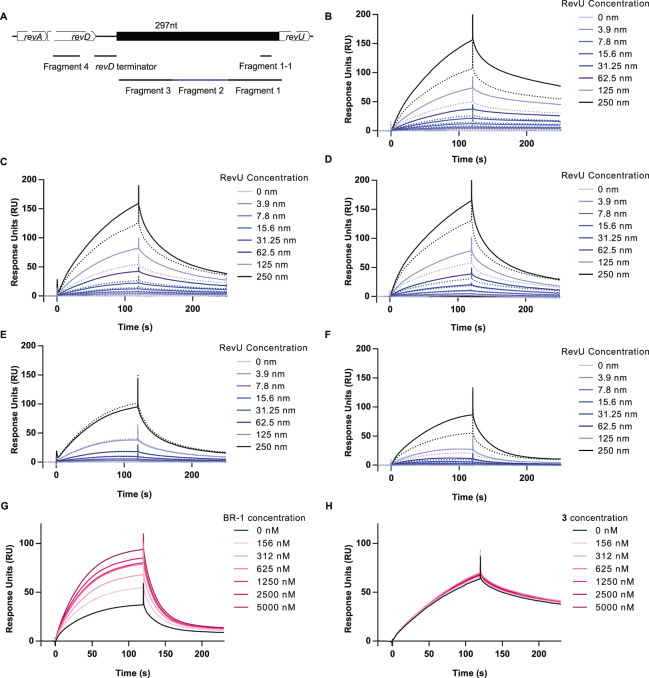
SPR analysis of the DNA-binding activity of RevU in the presence of BR-1. (**A**) Schematic representation of the *revU* promoter region and DNA fragments used for evaluating the RevU promoter–BR-1 interaction. Fragments 1–3, 99 bp in length, are located immediately upstream of the *revU* start codon. Fragment 4 (50-bp) located on *revD* coding sequence (GATTATGCGTCGCATTCGGTGTTTGTGGAGTTGATCGAGGATCGGGTTCT) was used for negative control. The underlined sequences were also found in another part of *revA* and *revD* sequence). The *revD* terminator sequence (CACCCAGCCCTCCCGCGGGAGCCGCCCGGCTCCCGCGGAAGGCGCCCGCG) lies upstream of fragment 3. Fragment 1-1 (22 bp: ACGCCGCAACGACCAACAGAGG) contains the putative lux-box sequence. Blank-subtracted SPR sensorgram showing the binding of biotin-labelled DNA fragment 1 (**B**), fragment 2 (**C**), fragment 3 (**D**), fragment 4 (**E**), and fragment 1-1 (**F**) to RevU in the presence (solid lines) or absence (dotted lines) of 1.25 µM BR-1. RevU was injected at various concentrations across the chip surface. Blank-subtracted SPR sensorgram showing the binding of biotinylated DNA fragment 1-1 to RevU (125 nM). Various concentration of BR-1 (**G**) and **3** (**H**) were tested. Fold changes in response unit BR-1 (+/−) is shown in Fig. S5.

Here, we showed that *revU* gene expression was enhanced after BR-1 treatment, and BR-1 specifically bound to RevU. Based on all of the observations (Figs. 1–4 and S1–S4), we hypothesized that BR-1 binding to RevU might facilitate binding to the *revU* gene promoter and activate *revU* gene expression. To examine whether RevU binding to its promoter region is enhanced by BR-1, we immobilized biotin-labelled, double-stranded DNA fragments on a sensor chip with streptavidin and evaluated RevU-promoter binding activity via SPR. Although RevU bound to all DNA fragments in a concentration-dependent manner, the presence of 1.25 µM BR-1 enhanced the binding activity of RevU to its promoter (Fragment 1–3), but not to another DNA region (Fragment 4) (Figs. 5A–E and S5). Moreover, it is reported that LuxR family regulators positively control secondary metabolism by binding to a conserved 20-bp lux-box CTG-(N_10_)-CAG in which five of the six bases are essential for LuxR binding^33^. Upstream of the *revU* gene, we found a candidate sequence referred to here as fragment 1–1. SPR analysis showed that RevU bound fragment 1-1 in a concentration-dependent manner and the binding was enhanced in the presence of 1.25 µM BR-1 (Fig. 5F). To determine the minimum effective concentration, various concentrations of BR-1 were run over the fragment 1-1-containing chip. We found that 156 nM BR-1 facilitated the DNA binding activity of RevU and the EC_50_ of BR-1 was 182 nM (Fig. 5G). The EC_50_ value was about half the concentration required for inducing RM production (0.35 µM)^28^. We have found that the tetrahydro form of BR-1 (**3)** did not induce the production of RMs^28^. Consistently, **3** did not enhance RevU binding to DNA (Fig. 5H). Taken together, these data suggested that activation of the *revU* gene was triggered by the binding of BR-1 to RevU. Interestingly, we could not find a lux-box sequence in Fragment 2 and 3. An exploration and detail characterization of RevU binding sequence will be our future study.

Moreover, to elucidate whether RevU-BR-1 complex directly enhanced the expression of *rev* structural genes or not, we searched for lux-box like sequences^33^ in each operon of *rev* cluster and found putative sequences in the upstream regions of *revA*, *revB*, *revJ*, *revK*, *revT*, and *revU* genes (Fig. S6A). We evaluated the binding of RevU to these regions in the presence and absence of BR-1using Bio-Layer Interferometry (Fig. S6B–H). We found that the addition of BR-1 resulted in enhanced binding of RevU to three putative lux-boxes for *revB*, *revK*, and *revU* genes. On the other hand, the enhanced RevU binding was not observed for *revA*, *revJ*, and *revT* genes, suggesting that *revB*, *revK*, and *revU* are mainly regulated through RevU.

### β-carbolines enhanced SM biosynthesis on *Streptomyces* sp. RK95-74 and *Streptomyces* sp. RK10-A626

We speculated that BR-1 and its derivatives might have an ability to induce SM in other *Streptomyces*. To test this hypothesis, *Streptomyces* sp. RK95-74 was cultured in the presence or absence of BR-1 or its derivatives. Interestingly, we found enhanced production of neomediomycin B, with a molecular mass of 1193 from biomediator treated *Streptomyces* sp. RK95-74 (Fig. S7)^34^. Similar to *Streptomyces* sp. SN-593^28^, the optimum β-carboline structures responsible for biomediator activity varied (Fig. S8A–C). We also cultured other *Streptomyces* spp. in the presence of β-carbolines and analysed the metabolites profiles using LC-MS. Treatment of *Streptomyces* sp. RK10-A626 with BR-1 resulted in the enhanced production of unidentified SM with *m/z* ratios ranging from 1032 to 1291 (Fig. S8D).

We also tested the production of stambomycins from *Streptomyces ambofaciens* in the presence of BR-1 or its derivatives because stambomycins from *S. ambofaciens* were obtained by expression of a LuxR family regulator (SamR0484) associated with the sleeping gene cluster^35^. However, the treatment of BR-1 derivatives did not result in the production of stambomycins. The alignment amino acid sequences between SamR0484 and RevU showed 34% identity and 47% similarity with a 7% gap, and phylogenetic analysis indicated that SamR0484 did not clade with RevU (Fig. S9). Because of the structural diversity of LuxR regulators, we expect that β-carbolines chemical signals are functional for a limited number of *Streptomyces* strains.

## Discussion

Understanding of chemical signals and the related mode of action is of profound importance. In this paper, we discovered that the β-carboline compound (BR-1) enhanced the production of RMs from *Streptomyces* sp. SN-593 through selective activation of the *rev* gene cluster (Fig. 2). Based on *revU* gene disruption and RT-qPCR analysis, we found that RevU, LuxR family regulator, regulates structural genes involved in RM-A biosynthesis (Figs. 3 and S2). Moreover, BR-1 enhances RevU binding to its own promoter and leads to self-activation of *revU* gene expression (Fig. 5). We also demonstrated that RevU-BR-1 complex is involved in the enhanced expression of *rev* structural genes such as *revB* and *revK*, but not for *revA*, *revJ*, and *revT* genes (Fig. S6).

RevU contains an N-terminal, ATP-binding region with Walker A and B motifs and a C-terminal DNA-binding region (helix-turn-helix domain)^25^. LuxR family regulators found in Gram-negative bacteria respond to acyl-homoserine lactone-type compounds, and ligand binding at their N-terminal regions facilitates their dimerization^36^. In the case of *Streptomyces* sp. SN-593, the binding of BR-1 to RevU may also cause structural changes or dimerization to facilitate RevU binding to its promoter and accelerate gene expression. In this paper, we also showed that BR-1 enhanced the production of SMs from *Streptomyces* sp. RK95-74 and *Streptomyces* sp. RK10-A626 (Figs. S7 and S8). To elucidate a mode of action of BR-1 in these strains, genome sequence, transcriptome analysis after treatment of BR-1, and gene disruption of transcriptional regulators will be our future study. Moreover, it is of interest to perform extended screening of actinomycetes which show the ability to enhance SM production by treatment of BR-1 and its derivatives because there are many RevU homologs in the database (Fig. S9).

Natural soil environments consist of diverse plants and microbial communities, where the presence of a wide range of chemical communications may trigger a variety of responses, including the production of bioactive molecules. For instance, in response to flavonoids released from plant roots, *Rhizobium* induces the expression of *nod* genes encoding enzymes for Nod factors^37^. Then, it induced the development of nodules in plant roots to promote growth. Plants provide a variety of organic compounds derived from photosynthesis in the rhizosphere^38,39^, a process known as rhizodeposition. Subsequently, microorganisms may provide benefits for plant growth^40^. β-carboline alkaloids widely found in plants are also isolated from fungi, marine tunicate, and actinomycetes^41–43^. However, their physiological roles in nature are not well understood. Interestingly, RMs produced from *Streptomyces* sp. SN-593 in response to β-carboline treatment have the antifungal activity against plant pathogenic fungi^22,44^. The metabolites may protect the producing strain itself and/or plants. In this study, our findings might shed light on hidden roles of β-carboline in nature. Searching for natural counterparts of β-carboline alkaloids and their relationship with symbiosis will be the subjects of future studies.

To summarize, we discovered that β-carboline-based chemical signals enhanced the production of SMs in some *Streptomyces* spp. In the case of *Streptomyces* sp. SN-593, we demonstrated that the activation of a LuxR family regulator was responsible for the phenomena. In general, LuxR regulators found in Gram-negative bacteria respond to acyl-homoserine lactone. Data generated in this study suggested that previously unknown interactions occur between β-carboline compounds and the LuxR regulator in *Streptomyces* sp. SN-593, a Gram-positive bacterium. Hidden chemical communication signals similar to β-carboline might act to induce secondary metabolites and our study may open avenues for enhanced production of natural products without genetic engineering.

## Methods

### Bacterial strains, plasmids, and culture medium

The bacterial strains and plasmids used are shown in Table S3. Luria–Bertani broth (Nacalai Tesque, Japan) was used to culture *E. coli*. The media were supplemented with appropriate antibiotics (100 µg ml^−1^ ampicillin, 50 µg ml^−1^ kanamycin, 50 µg ml^−1^ streptomycin, 100 µg ml^−1^ spectinomycin; 30 µg ml^−1^ chloramphenicol; 50 µg ml^−1^ ribostamycin; 5 µg ml^−1^ carumonam). Spores of *Streptomyces* species were prepared on MS plates and cultured using several media, including synthetic medium^45^, SK2^24^, MS^24^, RM high producing medium (RM-PM)^24^, YMG^46^, SY^46^, and RM low producing medium (SY-B) (1% soluble starch and 0.1% yeast extract).

### Metabolite profiles of RMs from *Streptomyces* sp. SN-593

Wild-type *Streptomyces* sp. SN-593, the Δ*revU* mutant, and strains transformed with pTYM*-*P_*aph*_-*revU* (Table S3) were cultured in SY medium (70 ml) at 28 °C in a cylindrical 500-ml flask at 150 rpm for ~2 days. The pre-culture medium was supplemented with kanamycin (1 µg ml^−1^) or thiostreptone (25 µg ml^−1^). One-millilitre cultures were added to RM-PM (70 ml) in 500-ml cylindrical flasks and cultured at 28 °C for 5 days. RM produced in the culture broth was extracted in an equal volume of acetone. After sonication and centrifugation at 5,000 × *g* for 10 min, the supernatant (8 ml) was concentrated to remove the acetone. The pH of the aqueous extract was adjusted to 4 with acetic acid and extracted twice with an equal volume of ethyl acetate. The dried residue was dissolved in methanol (1.2 ml), and 1 µl was analysed by LC-MS.

### Culture conditions and metabolite extraction from *Streptomyces* species after treatment with β-carboline compounds

*Streptomyces* sp. SN-593, the Δ*revU* mutant, and *Streptomyces* sp. RK10-A626 were cultured in 70 ml SY medium at 28 °C for 2 days. One ml of each culture was diluted in 100 ml SY-B medium, and 1-ml aliquots were cultured in 2.2-ml wells of a 96-well plate (4titude, reorder # 4ti-0130) for 3 days in the presence or absence of 1 μg ml^−1^ β-carboline compounds. *Streptomyces* sp. RK95-74 was cultured for 2 days at 28 °C in 70 ml of YMG medium. Then, 1 ml culture was diluted into 100 ml of YMG medium. The 1-ml aliquots were cultured in separate 2.2-ml wells for 3 days in the presence or absence of β-carboline compounds. Subsequently, the broth was mixed with 0.5 ml acetone, sonicated, and centrifuged at 36,200 × *g* for 10 min. Metabolites in the supernatant were analysed by LC-MS or UPLC-MS. The treatment of 1 μg ml^−1^ carboline compounds had no obvious effect on cell growth^28^.

### LC-MS analysis

Analysis of metabolites from *Streptomyces* sp. SN-593, *Streptomyces* sp. RK95-74, and *Streptomyces* sp. RK10-A626 was performed by ESI-MS using a Waters Alliance high-performance liquid chromatography (HPLC) system equipped with a mass spectrometer (Q-Trap; Applied Biosystems)^24^. The HPLC system consisted of an XTerra^®^MSC18 (5-μm, 2.1 mm internal diameter × 150 mm length) column maintained at 0.2 ml min^−1^. Solvent A was 0.05% aqueous formic acid and solvent B was acetonitrile. The sample was injected into the column after pre-equilibration with 30% solvent B; the column was developed with a linear gradient from 30% to 100% solvent B over 20 min and maintained in 100% solvent B for 20 min. Mass spectra were collected in ESI-negative and -positive modes. Analysis of metabolites from *Streptomyces* sp. RK95-74 was also performed by UPLC/ESI-MS analysis. An API3200 (Applied Biosystems, CA, USA) was connected to a Waters ACQUITY UPLC-H-class instrument with a photodiode-array detector (Waters, MA, USA) and an ACQUITY UPLC® BEH C18 (1.7-µm, 2.1-mm internal diameter × 50 mm length; Waters). The elution conditions were as follows: *Streptomyces* sp. RK95-74, 0.6 ml min^−1^; solvent A: water containing 0.05% formic acid; and solvent B: acetonitrile. After sample injection into a column equilibrated with 30% solvent B, the column was developed with a linear gradient of 30–100% solvent B over 2.5 min and maintained at 100% solvent B for 5 min.

### Chemical synthesis of β-carboline derivatives

All the β-carboline derivatives used in this manuscript are listed in Figs. 1 and S8A. The synthesis and confirmation of the structure was performed as explained^28^.

### Genome-sequencing analysis

DNA isolation and manipulation were performed as described^47^. Genomic DNA was isolated from *Streptomyces* sp. SN-593 and genome-sequence analysis was performed as described^24^. Briefly, the genomic DNA was cloned into vectors with an average insert size of 2–5 kb and 40 kb, respectively. End sequencing was performed using BigDye terminator 3.1 (Applied Biosystems). Sequencing product were analysed on an automated 3730 × 1 capillary sequencer (Applied Biosystems). Low-quality regions in the assembly were resequenced.

### RNA-sequence analysis

*Streptomyces* sp. SN-593 was cultured in the presence or absence of 3.5 µM BR-1. After 1 day, cultures from 8-well plates were harvested by centrifugation at 5,000 × *g* for 10 min. Total RNA was isolated using the RNeasy Protect Bacteria Mini Kit (Qiagen). The integrity and purity of the RNA was analysed using an Agilent Bioanalyzer. Five micrograms of total RNA was subjected to rRNA depletion using the RiboZero Meta-Bacteria Kit (Epicentre Biotechnologies, Madison, WI, USA), and Complementary DNA (cDNA) was generated using the NEBNext mRNA Sample Prep Kit (New England Biolabs, Ipswich, MA USA). cDNA was subjected to Illumina library preparation using NEBNext reagents (New England BioLabs, Ipswich, MA USA). The quality, quantity, and size distribution of the Illumina libraries were determined using an Agilent Bioanalyzer 2100. The libraries were submitted for Illumina HiSeq 2000 sequencing following a standard protocol. Paired-end 90- or 100-nucleotide reads were generated, and the data quality was checked using FastQC software (Babraham Institute, Cambridge, UK). The obtained data were analysed using CLC Genomics Workbench Server (Qiagen). Reads per kilobase per million mapped sequence reads (RPKM) values were used to determine gene-expression levels. Fold-expression levels were calculated as the RPKM in the presence of BR-1/the RPKM in the absence of BR-1.

### Quantitative PCR (qPCR) analysis

Total RNA was isolated using the RNeasy Protect Bacteria Mini Kit (Qiagen). RNA samples were treated with the RNase-Free DNase Set (Qiagen) and the RNeasy MinElute Cleanup Kit (Qiagen). The concentration and RNA integrity number (average: 8) were checked using an Agilent Bioanalyzer 2100. First-strand cDNA was synthesized using random hexamers and Superscript III reverse transcriptase (Invitrogen), and the cDNA was subsequently diluted with 5 volumes of nuclease-free water. qPCR amplification mixtures contained template cDNA, SsoFast™ EvaGreen® Supermix (BioRad), and forward and reverse primers (Table S4). Reactions were run on a CFX96 Real-Time PCR Analysis System (BioRad). Thermocycling proceeded at 95 °C for 30 s and 40 cycles at 95 °C for 5 s and 61 °C for 10 s.

### Gene disruption and complementation of *revU*

To construct the *revU* gene-disruption plasmid, a 7,784-bp region encoding the *revU* gene was amplified from pCC1FOS^TM^ clone (30B04)^24^ using the primers, 5′-CCCAAGCTTGGATCACCTTGCTGGACCTTGGTG-3′ and 5′-CCCAAGCTTGGAACGCGGTCCTGACGATCGAGC-3′. The underlined sequences indicate *Hind*III site. After enzyme digestion, the insert was ligated into the *Hind*III site of the pIM vector^46^ to construct pIM-*revU*. To create the disruption cassette, the *aphII* gene was amplified from the pKD13 plasmid^48^ using the primers, 5′-GCGGCGATGAAGATCCTCACCGAGTCCCGGCTGATCGAGATTCCGGGGATCCGTCGACC-3′ and 5′-GATCTCGACGCCCCATGCCTCCACCGACATACTGCGCAGTGTAGGCTGGAGCTGCTTC-3′). The underlined text (39-bp) show sequences homologous to the target-DNA region. The *revU* gene was replaced by the *aphII* gene using λ-red-mediated recombination to construct the gene-disruption plasmid pIM-Δ*revU*. The vector was then transformed into *Streptomyces* sp. SN-593 by conjugal gene transfer^24,49^. Gene disruption was confirmed by Southern hybridization. To complement the gene disruption, the *revU* gene was amplified from the 30B04 fosmid clone using the primers, 5′-CGCGGATCCATGCTGGAGCGCGACCAATGCCTC-3′ and 5′-CCCAAGCTTTCAGGACGCGAGGCTGGCCGGCA-3′. The underlined sequences indicate *Bam*HI and *Hind*III sites, respectively. After enzyme digestion, the insert was ligated into the *Bam*HI/*Hind*III sites of pTYM*-*P_*aph*_ to construct pTYM*-*P_*aph*_-*revU*. The plasmid was introduced into the *revU* gene disruptant and wild-type *Streptomyces* sp. SN-593. The selection of exconjugants was performed on SY agar plates containing 25 µg ml^−1^ thiostreptone and 5 µg ml^−1^ carumonam.

### Southern hybridization

Thirty micrograms of genomic DNA was digested with 50 units *Nco*I. The digested DNA (1 μg) was loaded onto a 0.9% agarose gel, run in Tris-acetate-ethylenediaminetetraacetic acid (EDTA) buffer, and stained with ethidium bromide. The DNA was then transferred to a Hybond N^+^ membrane (GE Healthcare) and fixed by baking overnight at 80 °C. The DNA probe was amplified from the gene-disruption plasmid (pIM*-*Δ*revU*) by PrimeSTAR^®^HS DNA polymerase (TaKaRa) at 98 °C for 10 s; 25 cycles of 98 °C for 10 s, 65 °C for 5 s, and 68 °C for 3 min; and a final extension at 68 °C for 6 min, using the oligonucleotide primers RevU-F (5′-ATGCTGGAGCGCGACCAATGC-3′) and ORF1-F (5′-ATGGTCCGCGAACTCGCGTAC-3′). The amplified DNA (4,249 bp) was purified by QIAquick^®^ Gel Extraction Kit (Qiagen) and labelled for Southern hybridisation with AlkPhos Direct Labeling Reagents (GE Healthcare). The hybridized DNA was detected using CDP-Star^TM^ reagent (GE Healthcare). The chemiluminescent signal on the membrane was exposed to X-ray film (Kodak).

### Vector construction, transformation, and expression of RevU in *S. lividans* TK23

The *revU* gene was amplified from the 30B04 fosmid clone using PrimeSTAR^®^HS DNA polymerase (TaKaRa) and thermocycling at 98 °C for 10 s and 25 cycles of 98 °C for 10 s, 62 °C for 5 s, and 68 °C for 1.5 min. The amplification primer sequences were: 5′-GGAATTCCATATGCTGGAGCGCGACCAATGCCTC-3′ and 5′-CCGCTCGAGTCAGGACGCGAGGCTGGCCGGCA-3′. The underlined sequences indicate *Nde*I and *Xho*I sites, respectively. After enzyme digestion, the insert was ligated into the pET28b(+) vector to construct pET28b(+)-*revU*. The β lactamase gene in the pWHM3 vector was replaced with the *aphII* gene using λ Red recombination to form the pWK vector. Then, the *tipA* promoter (P_*tipA*_) was inserted into the *Eco*RI and *Bam*HI sites to construct pWK-P_*tipA*_^50^. To facilitate protein purification, the *revU* gene containing a His-tag was amplified from pET28b (+)-*revU*. The primers used were 5′-CGCGGATCCATGGGCAGCAGCCATCATCAT-3′ and 5′-CCCAAGCTTAGCCGGATCTCAGTGGTGGTG-3′. The underlined sequences indicate the *Bam*HI and *Hind*III sites. After enzyme digestion, the insert was ligated into pWK-P_*tipA*_ to generate pWK-P_*tipA*_-*revU*. The vector was introduced into *S. lividans* TK23 using a polyethylene glycol-mediated protoplast method^51^. The resulting transformants were grown in 70 ml SK2 medium in a 500-ml cylindrical flask at 28 °C at 150 rpm. RevU expression was induced in the presence of 50 μg ml^−1^ thiostrepton. Cells were harvested by centrifugation at 5,000 × *g* for 10 min and sonicated in 5 ml binding buffer (50 mM Tris-HCl [pH 7.5], 500 mM NaCl, 10% glycerol, and 5 mM imidazole) containing a protease inhibitor cocktail (Roche). The cell lysate was centrifuged at 36,200 × *g* for 10 min at 4 °C, and the supernatant was used for BR-1 bead-binding experiments.

### *revU* gene expression in *E. coli* and purification

The synthetic *revU* gene (*revUsyn*) in the pMA-RQ vector (Life Technologies) was ligated into the *Nde*I and *Xho*I sites of pColdI (TaKaRa) to create pColdI-*revUsyn*, and transformed into *E. coli* BL21 cells. The cells were cultured overnight in LB medium containing 100 µg ml^−1^ ampicillin. The preculture was added to 100 ml of LB-ampicillin (100 µg ml^−1^) medium in a 500-ml cylindrical flask. The cells were cultured at 37 °C until the OD_600_ reached 0.2. The cells were cooled on ice for 30 min, and isopropyl β-D-1-thiogalactopyranoside was added at to 0.5 mM. The cells were incubated at 18 °C for 24 h and collected by centrifugation at 5,000 × g for 10 min. Purification of the His6-tagged RevU protein was performed as described^46^. The purity was confirmed by 5–20% SDS-PAGE.

### BR-1 chemical bead preparation and protein binding detection

BR-1 and control beads were prepared as described^31,32^. Briefly, 100 μl of photoaffinity linker-coated agarose beads was suspended in 200 μl of isopropyl alcohol and dried in vacuo. Methanol (control beads) or BR-1 solution (1 mg/150 μl methanol) was added, and the mixture was dried in vacuo. The beads were irradiated at 365 nm using an ultraviolet (UV)-activated crosslinker (4 J/cm^2^). The irradiated beads were sequentially washed in 50% methanol, methanol, DMSO, and methanol (3 × 400 μl/wash) and suspended in 200 μl PBS (1.8 mM KH_2_PO_4_, 10 mM Na_2_HPO_4_•12H_2_O, 137 mM NaCl, and 2.7 mM KCl). To examine protein binding, 25 µl control beads or BR-1-conjugated beads was mixed with cell lysates (2 mg protein) from *S. lividans* TK23 cells expressing the RevU protein and incubated overnight at 4 °C in 1 ml binding buffer. Then, the beads were washed 5 times with binding buffer containing 0.1% Tween-20. The bound proteins were eluted with sodium dodecyl sulphate-polyacrylamide gel electrophoresis (SDS-PAGE) sample buffer. The eluted protein was analysed by 7.5% SDS-PAGE. To identify proteins, MALDI-TOF/MS analysis was performed using an Ultraflex instrument (Bruker Daltonics).

### Competition assay

The purified His6-tagged RevU protein (100 ng) was dissolved in 500 µl binding buffer containing 20 μg ml^−1^ bovine serum albumin. The solution was mixed with 10 µl of control and BR-1 beads in the presence or absence of 2 µg free BR-1. After overnight incubation at 4 °C, the beads were washed 5 times with binding buffer containing 0.1% Tween-20, and the bound proteins were eluted in SDS-PAGE buffer. The eluted proteins were separated by 5–20% SDS-PAGE and transferred to a nitrocellulose membrane. The membrane was treated with a mouse anti-His antibody (GE healthcare) and a horseradish peroxidase-conjugated AffiniPure goat anti-mouse IgG (H + L) (Jackson ImmunoResearch). After treatment with Pierce^TM^ ECL Western Blotting Substrate (ThermoFisher), the membrane was exposed to X-ray film (Kodak).

### SPR analysis

SPR measurements were performed on a BIAcore T200 instrument (GE Healthcare) maintained at 25 °C. The SPR buffer (50 mM Tris-HCl [pH 7.5], 500 mM NaCl, and 0.05% (v/v) Tween-20) was used to evaluate BR-1 binding to RevU. The purified His6-tagged RevU protein was diluted to 5 μg ml^−1^ in running buffer and immobilized on a Series S Sensor Chip NTA (GE Healthcare) at 5 μl min^−1^ for 600 s, with a 600-s stabilization period. β-carboline compounds were diluted in SPR buffer and injected over the sensor chip for 60 s at 30 μl min^−1^. Dissociation proceeded for 120 s. RevU binding to the RevU promoter was measured in 10 mM HEPES (pH 7.4), 3 mM EDTA, 150 mM NaCl, and 0.05% (v/v) Tween-20. Double-stranded, 5′ biotinylated DNA was synthesized by Hokkaido System Sciences. The labelled promoter DNA was diluted to 30 nM in SPR buffer and immobilized on a Series S Sensor Chip SA (GE Healthcare), with immobilized streptavidin, at 15 μl min^−1^ for 180 s. RevU, dissolved in 10 mM HEPES (pH 7.4) and 3 mM EDTA, was diluted in SPR buffer. Various RevU concentrations (ranging from 3.9 nM to 250 nM) were injected over the surface for 120 s at 15 μl min^−1^. Dissociation occurred for 120 s. Regeneration of the SA sensor surface was performed with 2 M NaCl for 30 s at 30 μl min^−1^. The effect of BR-1 on RevU–RevU promoter binding was studied by injecting 1.25 µM BR-1 pre-mixed with various RevU concentrations. Sensorgrams were analysed using BiaEvaluation software (GE Healthcare), and affinity constants were calculated using sample values obtained by subtracting blank values.

### Bio-Layer Interferometry analysis

BLI measurements were performed on a BLItz system (ForteBio), a label-free technology that analyses the interference pattern of white light reflected from two surfaces, maintained at room temperature. The BLItz buffer contained 10 mM HEPES (pH 7.3), 3 mM EDTA (pH 8.0) (both diluted from 1 M concentrate); 150 mM NaCl, and 0.05% (v/v) Tween-20. The labelled promoter DNA was diluted to 200 nM in BLItz buffer and immobilized on a streptavidin biosensor, followed by 120 s each of association and dissociation with 125 nM RevU in the presence and absence of 5 µM BR-1.

## Supplementary information

SI.

## Data Availability

The data that support the finding of this study are available from the corresponding author upon request. Sequence data have been deposited to DNA data bank (accession number AP018365 RVR, AP018366 pRVR1, and AP018367 pRVR2). The RNA-seq data were deposited in SRA database with accession code SRR6715512 and SRR6715514. The data that support the finding of this study are available from the corresponding authors upon request.
