# Supplementary material for: β-carboline chemical signals induce reveromycin production through a LuxR family regulator in Streptomyces sp. SN-593
Source: Sci Rep. 2020 Jun 23;10:10230. doi: 10.1038/s41598-020-66974-y (PMC7311520; doi:10.1038/s41598-020-66974-y)
Supplement: Supplementary file 1 — SI. [file 41598_2020_66974_MOESM1_ESM.pdf]

Supporting Information

β-carboline chemical signals induce reveromycin production through a LuxR family regulator in *Streptomyces* sp. SN-593

Suresh Panthee[1,2], Naoko Kito[1], Teruo Hayashi[3], Takeshi Shimizu[3], Jun Ishikawa[4], Hiroshi Hamamoto[2], Hiroyuki Osada[3,*] and Shunji Takahashi[1,*]

Affiliations

[1] RIKEN Center for Sustainable Resource Science, Natural Product Biosynthesis Research Unit, Wako, Hirosawa 2-1, 351-0198 Saitama, Japan

[2] Teikyo University Institute of Medical Mycology, Otsuka 359, Hachioji, Tokyo, Japan

[3] RIKEN Center for Sustainable Resource Science, Chemical Biology Research Group, Wako, Hirosawa 2-1, 351-0198 Saitama, Japan

[4] Department of Bioactive Molecules, National Institute of Infectious Diseases, Toyama 1-23-1, Shinjuku, Tokyo 162-8640, Japan

* Correspondence and requests for materials should be addressed to H. O. hisyo@riken.jp; or S. T. shunjitaka@riken.jp; Tel: +81-48-467-9541; Fax: +81-48-462-4669.

Contents:

Figure S1. qPCR analysis of RM biosynthetic genes

Figure S2. Gene disruption of *revU*.

Figure S3. Display of full-length gels/blots presented in this manuscript.

Figure S4. Analysis of the RevU interaction with BR-1 and 3.

Figure S5. BR-1 facilitated the DNA-binding activity of RevU in the promoter region.

Figure S6. Analaysis of lux-box sequence in RM biosynthetic gene cluster.

Figure S7. High-resolution HPLC/MS analysis.

Figure S8. Effect of β-carboline compounds on *Streptomyces* spp.

Figure S9. Phylogenetic analysis of RevU homologs.

Table S1. **Summary of the fold-expression levels of 27 gene clusters from *S. reveromyceticus* SN-593 after BR-1 treatment.**

Table S2. MALDI-TOF/MS analysis of the BR-1-binding protein RevU

Table S3. Bacterial strains, plasmids, and DNA used in this study

Table S4. **Sequences of primers used for qPCR**

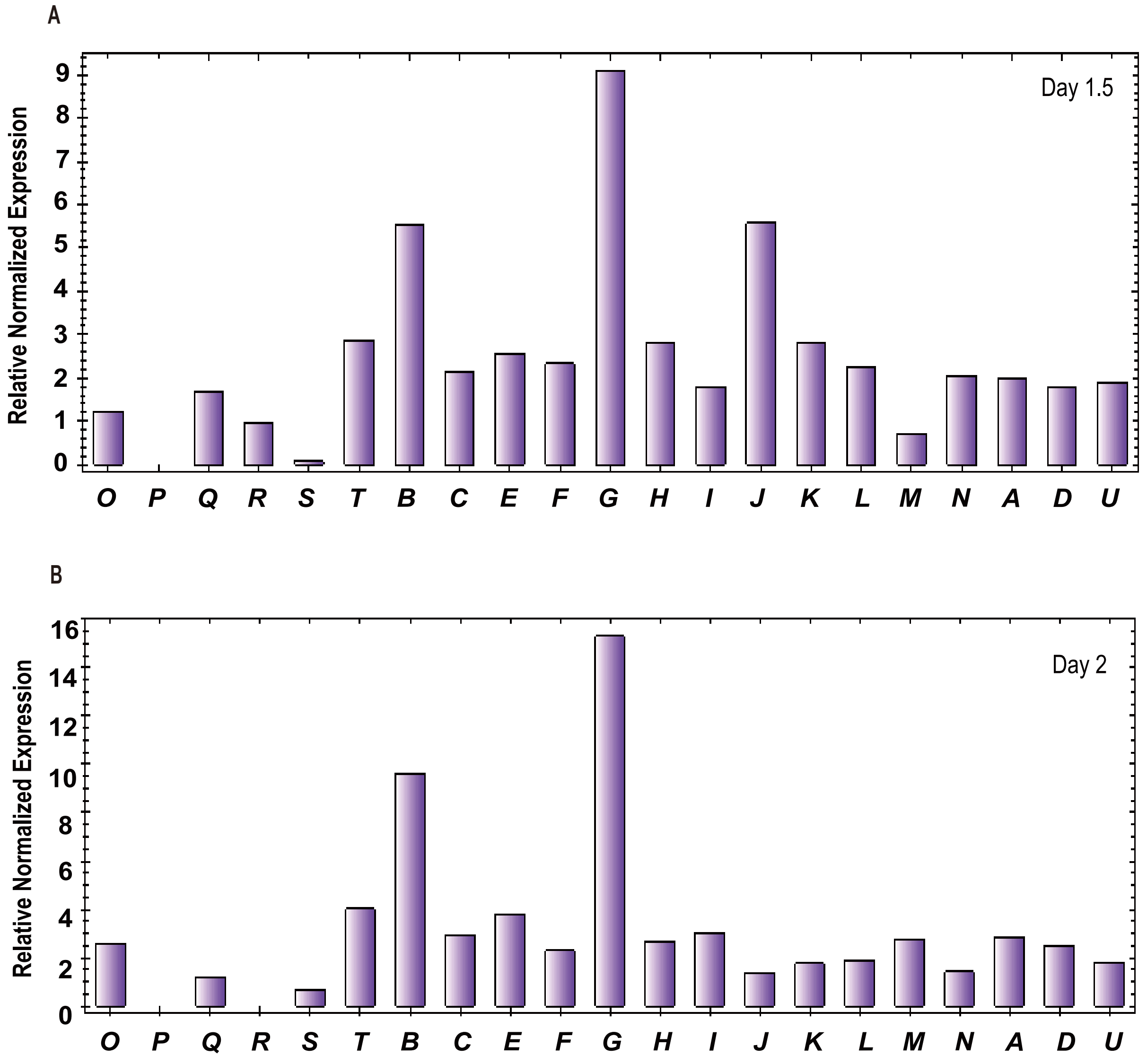

Figure S1. qPCR analysis of RM biosynthetic genes. *Streptomyces* sp. SN-593 was cultured in 10 ml of SY medium in a test tube at 28ºC in the presence or absence of 3.5 µM BR-1. mRNA was isolated at day 1.5 (A) and day 2 (B). Expression of the reference gene (*hrdB*) was measured as a control to normalise the differences in quantities of cDNA used as a template. The Y axis indicates the ratio of target-gene expression in BR-1-treated samples to that in samples not treated with BR-1, where the expression level of the target gene was divided by that of the *hrdB* gene. All data were analysed using CFX Manager software, version 3.1 (Bio-Rad).

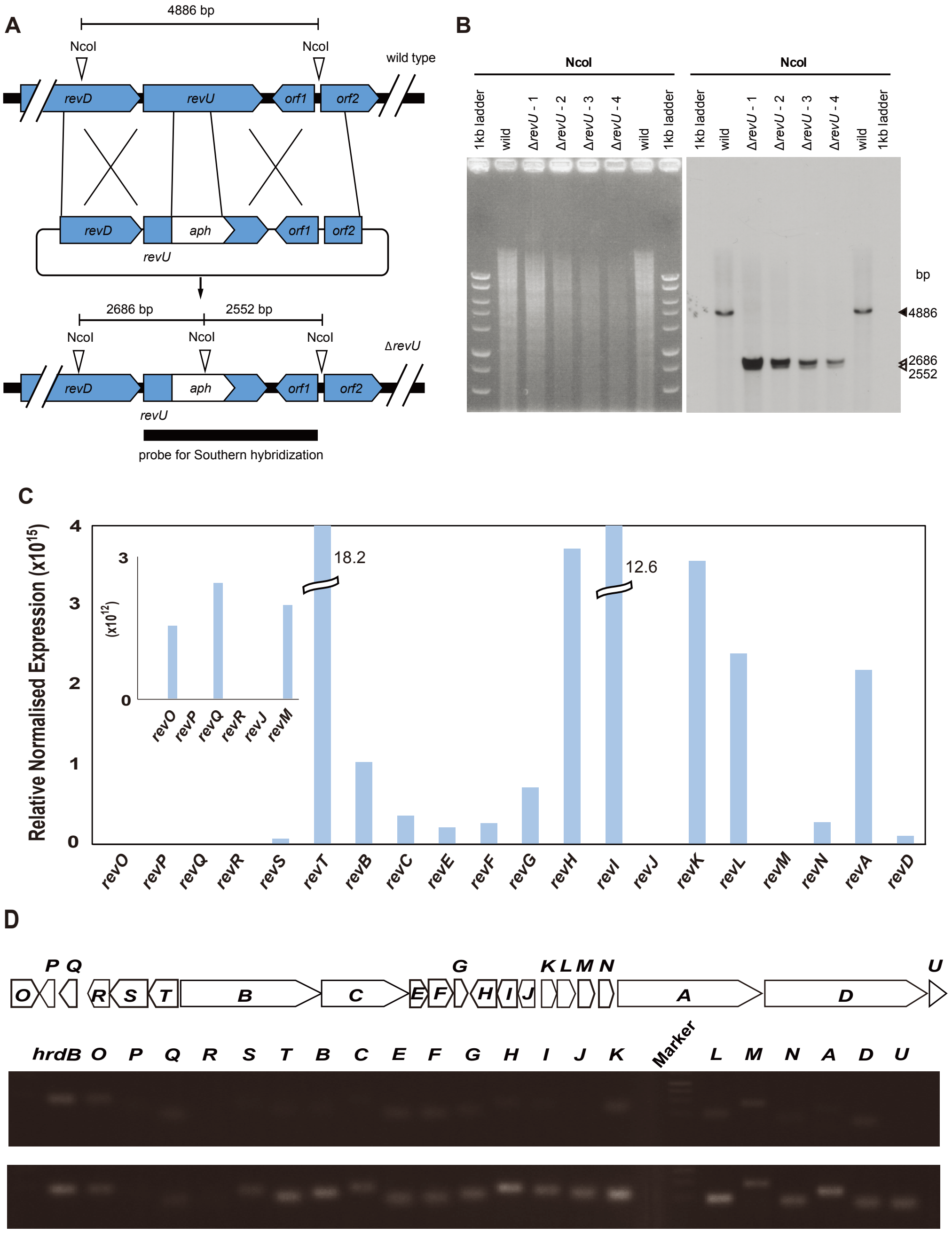

Figure S2. Gene disruption of *revU*. (A) Scheme for *revU* disruption and restriction maps of the wild-type gene and Δ*revU* mutant. The bar shows the expected fragment sizes (bp) following NcoI digestion (4886 bp for the wild-type gene, and 2552 and 2686 bp for the Δ*revU* mutant). (B) Southern hybridization analysis of wild-type (lanes 2 and 7) and Δ*revU* mutants (lanes 3, 4, 5, and 6; individual isolations) were performed using the probe shown in A. The arrows indicate the expected sizes of DNA fragments from the wild-type gene (solid) and

Δ*revU* mutant (open). (C) qPCR analysis of mRNA expression of genes involved in RM biosynthesis. Total RNA was extracted at day 2 from Δ*revU* and wild-type *Streptomyces* sp. SN-593 after culturing in 10 ml of SY medium. The reference gene (*hrdB*) was used as a control to normalise for differences in the quantity of cDNA used as a template. The Y axis indicates the ratio of target-gene expression in the wild-type sample to that in the Δ*revU* sample, where the expression level of the target gene was divided by that of the *hrdB* gene. All data were analysed using CFX Manager software, version 3.1 (Bio-Rad). (D) qPCR products after 40 cycles were run on a 1.5% agarose gel. Expression of *hrdB* is shown as the reference gene. Upper panel, organization of the RM biosynthetic gene cluster; middle panel, Δ*revU* mutant; lower panel, wild-type *Streptomyces* sp. SN-593.

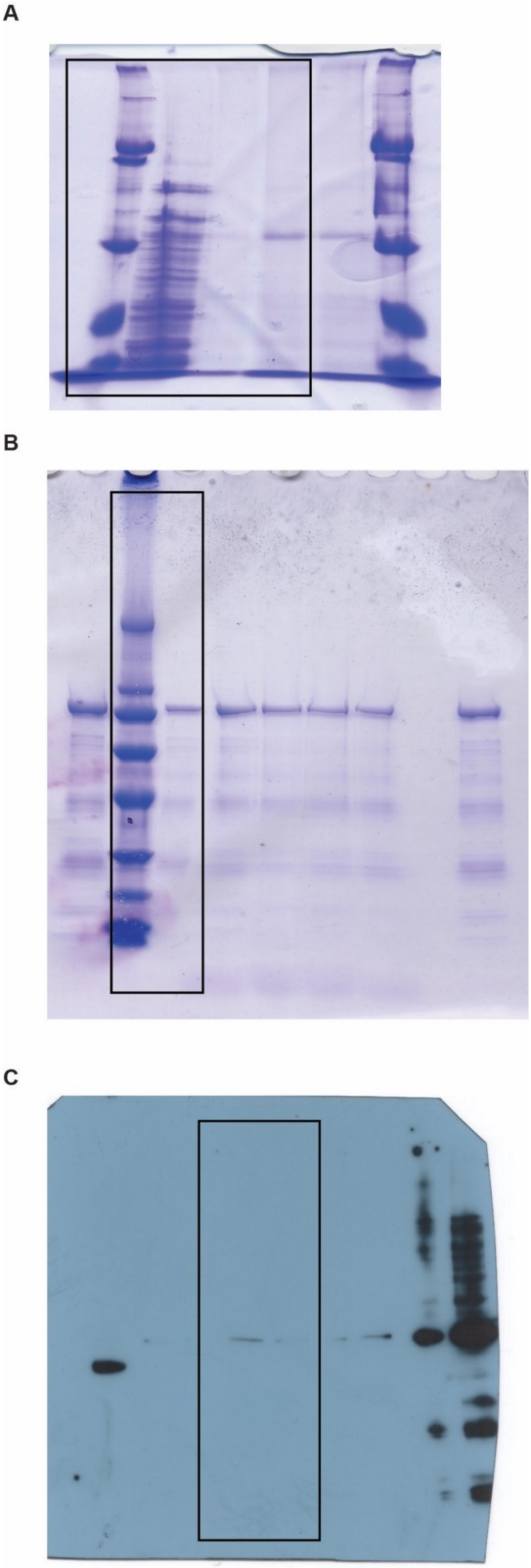

Figure S3. Display of full-length gels/blots presented in this manuscript. Uncropped images shown in Figure 4A (**A**), Figure 4B (**B**), and Figure 4C (**C**). The cropped region is indicated by box in each image

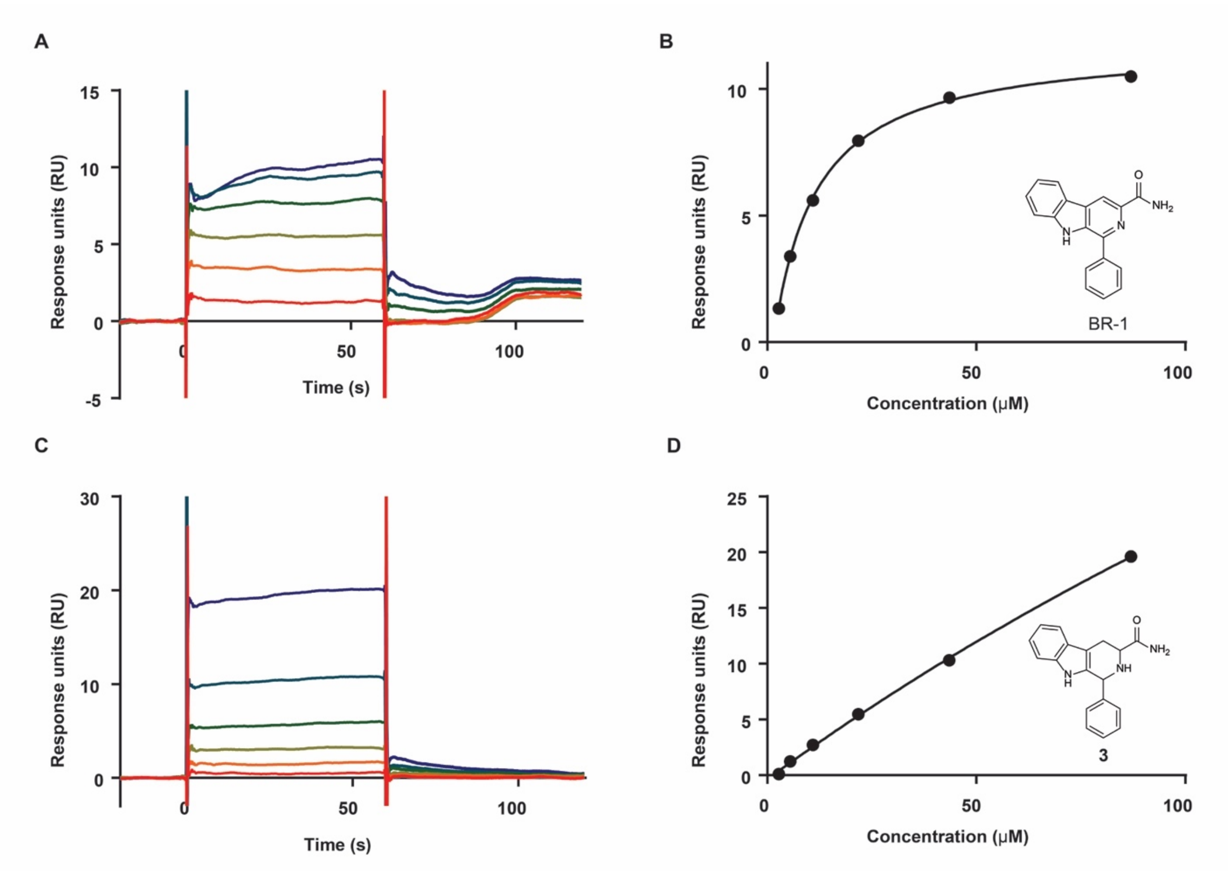

Figure S4. **Analysis of the RevU interaction with BR-1 and 3.** Representative blank-subtracted SPR sensorgram (A, C) and affinity curves for the interaction of BR-1 **(**B**)** and **3** (D) with RevU. Purified His6-tagged RevU was immobilized on a series S sensor chip NTA, and various concentrations of β-carboline compounds in SPR buffer were injected over the sensor chip. The concentrations of BR-1 and **3** used were 2.7, 5.4, 10.8, 21.7, 43.5, and 87 μM.

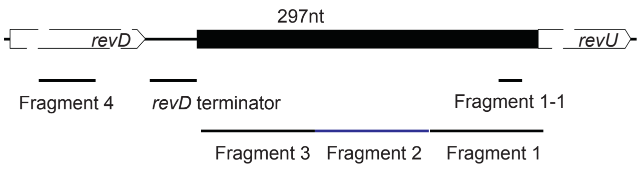

Fragment	Fold change in Response unit BR-1 (+/-)	BR-1 activity
Fragment 1	1.47 ± 0.01	+
Fragment 2	1.46 ± 0.16	+
Fragment 3	1.29 ± 0.05	+
Fragment 4	0.97 ± 0.05	-
Fragment 1-1	1.37 ± 0.17	+

Figure S5. BR-1 facilitated the DNA-binding activity of RevU in the promoter region. For each concentration of RevU analysed, the maximum response unit in the presence of 1.25 μM BR-1 was divided by the maximum response unit without BR-1 to calculate fold change **(related to Figure 5)**. Mean and standard deviation of fold changes at different concentrations (3.9 nM – 250 nM) for each fragmented was determined (n=7).

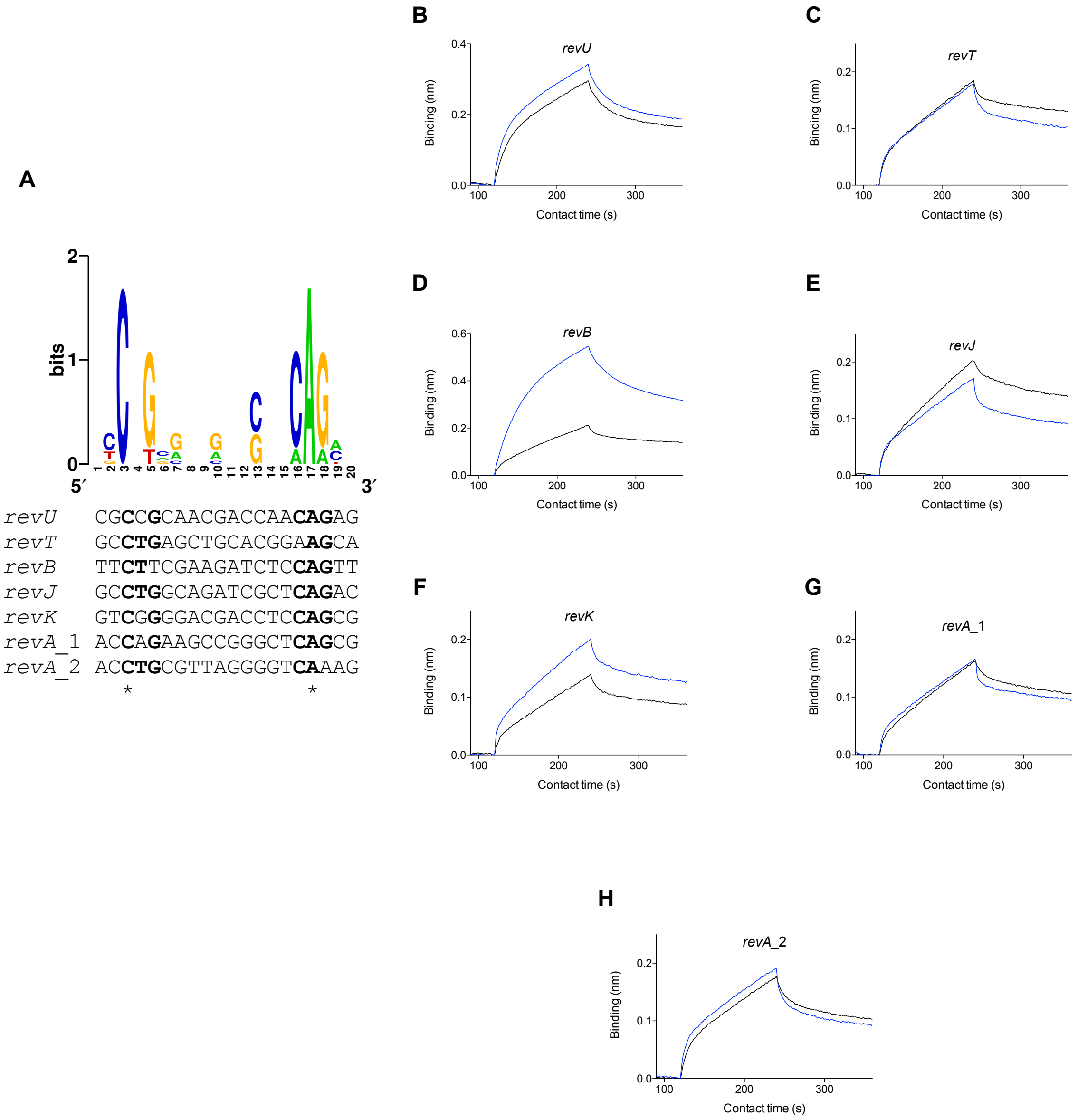

Figure S6. Analysis of lux-box sequence in RM biosynthetic gene cluster. (A) The sequence logo of the putative 20-bp lux-box sequence in the upstream regions of the RM biosynthetic operon constructed using WebLogo (http://weblogo.berkeley.edu/). (B-H) Bio-Layer Interferometry sensorgrams of the binding of 125 nM RevU to putative lux-box sequences of *revU*, *revT*, *revB*, *revJ*, *revK*, *revA*_1, and *revA*_2, in the presence (blue) and absence (black) of 5 µM BR-1, respectively. Biotinylated 22-bp double-stranded DNA that included 1-additonal nucleotide from both the upstream and downstream of the lux-box was synthesized by Hokkaido System Sciences. DNA was immobilized to streptavidin biosensor

using ForteBio's BLItz system and binding was measured using BLItz buffer that contained 10 mM HEPES (pH 7.3), 3 mM EDTA (pH 8.0) (both diluted from 1 M concentrate); 150 mM NaCl, and 0.05% (v/v) Tween-20.

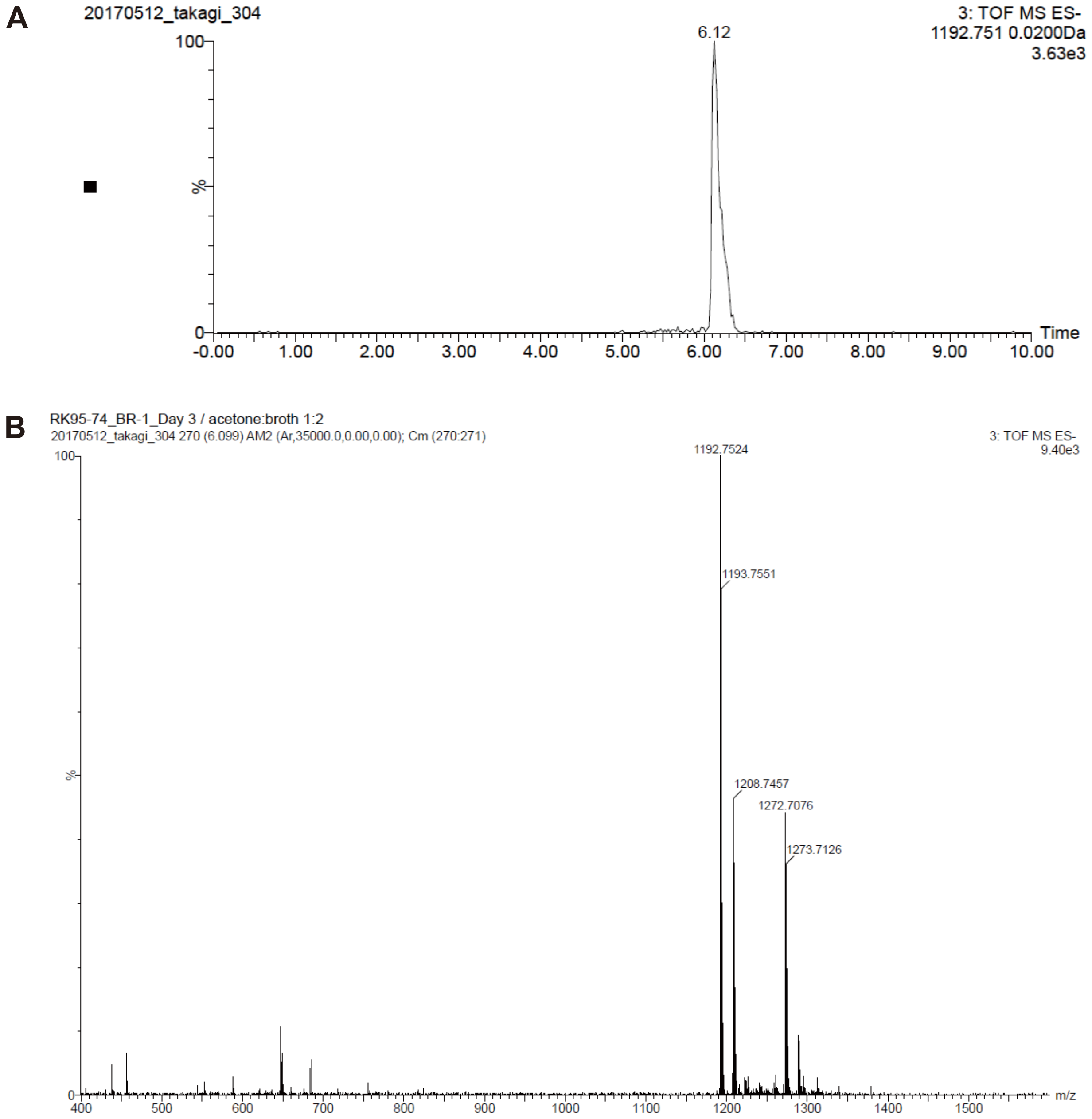

Figure S7. High-resolution HPLC/MS analysis. (A) Extracted ion chromatogram at an *m/z* ratio of 1192.741 – 1192.761 $[M - H]^-$. (B) High-resolution MS analysis of metabolite at a retention time of 6.12 min. After a *Streptomyces* sp. RK95-74 sample was cultured in the presence of BR-1 (1 µg ml^{-1}), metabolite production enhanced by BR-1 was subjected to high-resolution HPLC/MS analysis. ESI-TOF MS analysis was performed with a Synapt G2 mass spectrometer (Waters, Manchester, UK) equipped with an ACQUITY UPLC system (Waters).

The HPLC conditions were as follows: ACQUITY UPLC BEH C_{18} column (2.1 mm × 50 mm); flow rate of 0.2 ml min^{-1}; 0.1% formic acid in water as solvent A; 0.1 % formic acid in acetonitrile as solvent B; 10% B for 1 min, and then 10 – 80% B over 7 min.

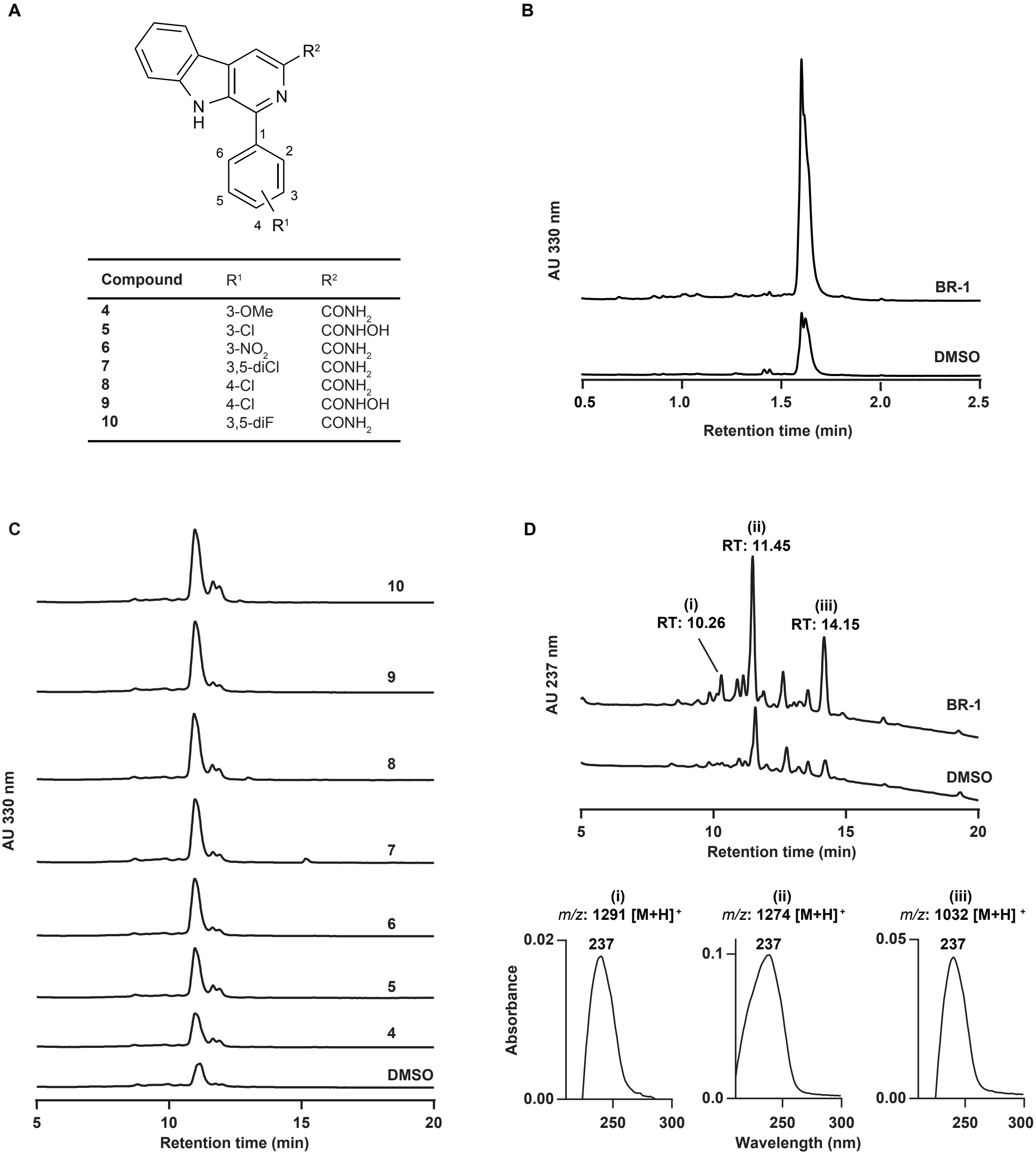

Compound	R^1	R^2
**4**	3-OMe	$CONH_2$
**5**	3-Cl	CONHOH
**6**	3-NO_2	$CONH_2$
**7**	3,5-diCl	$CONH_2$
**8**	4-Cl	$CONH_2$
**9**	4-Cl	CONHOH
**10**	3,5-diF	$CONH_2$

Figure S8. **Effect of β-carboline compounds on *Streptomyces* spp.** Structure of β-carboline derivatives (A)[1] and their effect on *Streptomyces* sp. RK95-74 (B, C) and *Streptomyces* sp. RK10-A626 (D). *Streptomyces* spp. were cultured in the presence or absence of 1 μg ml^{-1} β-carboline compounds. Metabolite profiles were analysed by UPLC-MS (B) or LC-MS (C, D). ESI-MS and UV spectra for the enhanced product peaks are shown (lower panel of D).

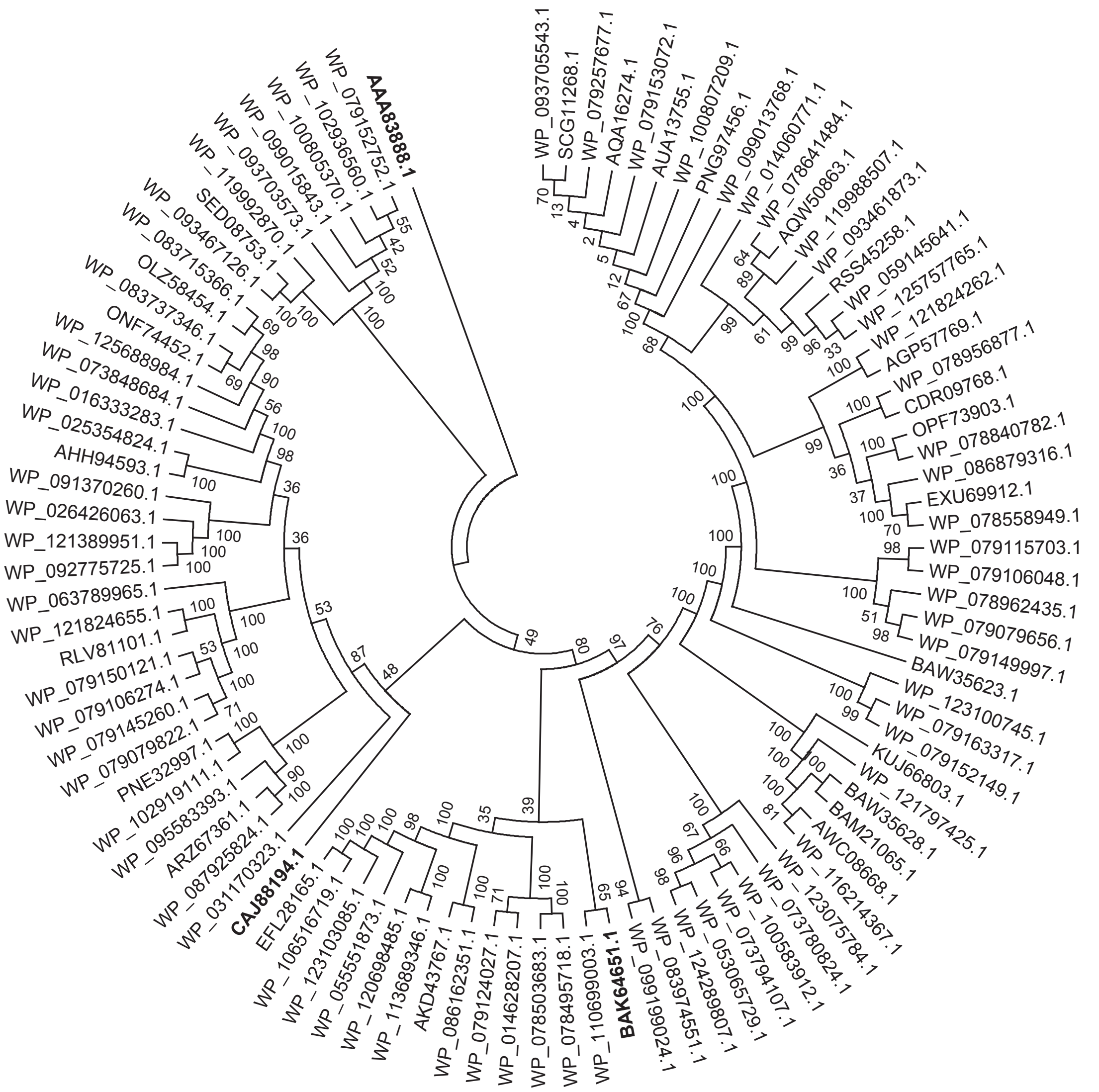

Figure S9. Phylogenetic analysis of RevU homologs. LuxR regulators which have a homology to RevU were obtained from NCBI using BLAST and analysed using MEGA7. The evolutionary history was inferred using the Neighbor-Joining method. Bootstrap values from 500 replicates are shown in the branches. The tree is drawn to scale, with branch lengths indidate the number of amino acid substitutions per site. MalT from *Escherichia coli* was used as an outgroup. The position of RevU (BAK64651.1), SamR0484 (CAJ88194.1), and MalT (AAA83888.1) is indicated by boldface characters. The accession number of RevU homologs are shown.

Table S1. Summary of the fold-expression levels of 27 gene clusters from *S. reveromyceticus* SN-593 after BR-1 treatment. T1PKS: type I polyketide synthase; T2PKS: type II polyketide synthase; T3PKS: type III polyketide synthase; NRPS: nonribosomal peptide synthetase; PKS/NRPS: polyketide synthase/nonribosomal peptide synthetase. Schematic representations of the gene organization including key secondary metabolite genes are presented.

Cluster 1. NRPS

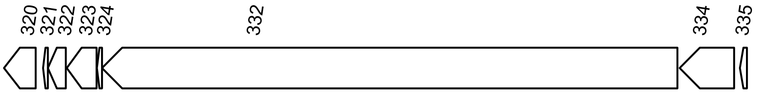

Gene	#AA	Putative function [blast hit species]	% identity	% positive	Accession Number	Fold-expression
RVR320	*441*	lipase [*Streptomyces* sp. MUSC 1]	72	85	WP_071383241	1.88
RVR321	*67*	hypothetical protein [*Streptomycetaceae*]	78	84	WP_049649157	1.00
RVR322	*252*	hypothetical protein [*Streptomyces aureofaciens*]	82	88	WP_033352041	1.03
RVR323	*415*	cytochrome P450 [*Streptomyces yeochonensis*]	87	93	WP_037906288	1.08
RVR324	*71*	mbtH-like protein [*Streptomyces* sp. CB03911]	91	97	WP_073928078	3.01
RVR332	*8024*	nonribosomal peptide synthetase [*Streptomyces olivochromogenes*]	81	87	WP_067380720	1.56
RVR334	*757*	ABC transporter [*Streptomyces* sp. ATexAB-D23]	81	87	WP_018550782	1.35
RVR335	*101*	hypothetical protein [*Kitasatospora* sp. MY 5-36]	75	86	WP_049651148	0.90

Cluster 2. T3PKS

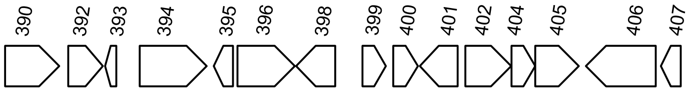

Gene	#AA	Putative function [blast hit species]	% identity	% positive	Accession number	Fold-expression
RVR390	*418*	histidine kinase [*Streptomyces yanglinensis*]	76	80	SEG92315	1.18
RVR392	*267*	DNA-binding response regulator [*Streptomyces yanglinensis*]	78	81	SEG92311	0.74
RVR393	*87*	hypothetical protein [*Streptomyces yeochonensis*]	74	93	WP_051951202	0.37
RVR394	*518*	esterase [*Streptomyces yanglinensis*]	49	62	SEG93046	4.14
RVR395	*149*	transcriptional regulator [*Nocardia vaccinii*]	69	79	WP_067899618	1.34
RVR396	*444*	aminomethyltransferase [*Nocardia vaccinii*]	84	91	WP_067899616	1.35
RVR398	*303*	hypothetical protein [*Streptomyces* sp. NRRL F-5126]	72	80	WP_030911152	1.05
RVR399	*180*	hypothetical protein [*Streptomyces* sp. NRRL F-5126]	82	88	WP_030911149	1.02
RVR400	*192*	acetyltransferase [*Streptomyces* sp. NRRL F-2799]	81	92	WP_030817356	0.87
RVR401	*293*	prenyltransferase [*Streptomyces paucisporeus*]	75	80	SHL42680	2.02
RVR402	*353*	polyketide synthase [*Streptomyces paucisporeus*]	72	79	WP_073495748	1.51
RVR404	*183*	methyltransferase [*Streptomyces yanglinensis*]	88	91	SEG56622	0.94
RVR405	*338*	oxidoreductase [*Streptomyces yanglinensis*]	84	88	SEG56637	3.02
RVR406	*540*	oxidoreductase [*Streptomyces yanglinensis*]	81	88	SEG84076	2.33
RVR407	*155*	polyketide cyclase/dehydrase [*Streptomyces yanglinensis*]	77	84	SEG86899	1.00

Cluster 3. T3PKS

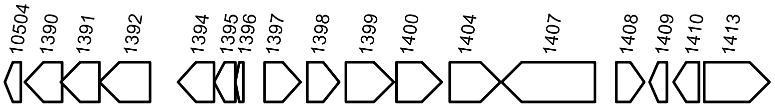

Gene	#AA	Putative function [blast hit species]	% identity	% positive	Accession number	Fold-expression
RVR10504	*128*	hypothetical protein [*Streptomyces tsukubensis*]	62	70	WP_077965690	1.15
RVR1390	*284*	hypothetical protein [*Streptomyces* sp. Amel2xE9]	95	98	WP_019985202	1.07
RVR1391	*301*	aldo/keto reductase [*Streptomyces caatingaensis*]	95	96	WP_049714342	1.01
RVR1392	*395*	hypothetical protein [*Streptomyces albus subsp. albus*]	96	97	KUJ64319	1.20
RVR1394	*277*	transcriptional regulator [*Streptomyces caatingaensis*]	95	97	WP_049714344	1.20
RVR1395	*168*	hypothetical protein [*Streptomyces caatingaensis*]	96	98	WP_049714345	0.76
RVR1396	*69*	hypothetical protein [*Streptomyces caatingaensis*]	95	95	WP_078870908	0.08
RVR1397	*276*	ABC transporter [*Streptomyces xylophagus*]	83	91	WP_051741765	1.65
RVR1398	*246*	ABC transporter [*Streptomyces* sp. URHA0041]	85	90	WP_033176034	1.57
RVR1399	*374*	NMT1/THI5 like protein [*Streptomyces violaceoruber*]	78	87	WP_030934103	1.43
RVR1400	*355*	oxidoreductase [*Streptomyces hokutonensis*]	88	93	WP_019073963	1.66
RVR1404	*394*	polyketide synthase [*Streptomyces autolyticus*]	82	88	WP_087684117	1.75
RVR1407	*721*	phosphatase [*Streptomyces rubidus*]	76	84	WP_079176053	1.33
RVR1408	*211*	hypothetical protein [*Streptomyces* sp. DvalAA-14]	56	68	SCD99018	0.00
RVR1409	*138*	hypothetical protein [*Streptomyces yanglinensis*]	74	84	SEG55314	2.53
RVR1410	*201*	hypothetical protein [*Streptomyces yanglinensis*]	55	64	SEG57500	1.35
RVR1413	*499*	major facilitator superfamily transporter [*Streptomyces yanglinensis*]	77	86	SEG57483	1.24

Cluster 4. Terpene

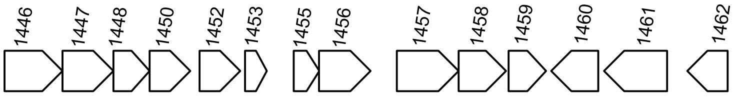

Gene	#AA	Putative function [blast hit species]	% identity	% positive	Accession number	Fold-expression
RVR1446	*450*	aminotransferase [*Streptomyces* sp. NRRL F-5123]	89	93	WP_031515564	1.67
RVR1447	*395*	dehydratase [*Streptomyces alni*]	94	96	SFF93754	1.23
RVR1448	*282*	amidohydrolase [*Streptomyces* sp. NRRL F-5123]	93	97	WP_031515559	1.72
RVR1450	*316*	dehydrogenase [*Streptomyces* sp. TSRI0281]	78	86	WP_073729555	0.81
RVR1452	*317*	acetyltransferase [*Streptomyces* sp. NRRL F-5123]	68	72	WP_031515553	0.00
RVR1453	*169*	hypothetical protein [*Streptomyces* sp. NRRL F-6602]	89	95	KPC88306	0.94
RVR1455	*198*	tetR family transcriptional regulator [*Actinomadura atramentaria*]	82	90	WP_019634167	1.41
RVR1456	*398*	hypothetical protein [*Streptacidiphilus albus*]	80	86	WP_034090968	2.09
RVR1457	*479*	transcriptional regulator [*Streptomyces* sp. CT34]	77	88	WP_043262806	1.61
RVR1458	*368*	terpene synthase [*Streptomyces* sp. AS58]	80	89	WP_053761854	1.53
RVR1459	*291*	geranyl diphosphate methyltransferase [*Streptomyces yanglinensis*]	91	95	SEG55734	1.54
RVR1460	*366*	hypothetical protein [*Streptomyces* sp. NRRL S-813]	73	82	WP_030164489	1.47
RVR1461	*491*	glycosyl hydrolase [*Streptomyces yanglinensis*]	87	90	SEG55752	1.55
RVR1462	*313*	aminoglycoside phosphotransferase [*Streptomyces lydicus*]	87	93	WP_046930280	1.48

Cluster 5. T2PKS

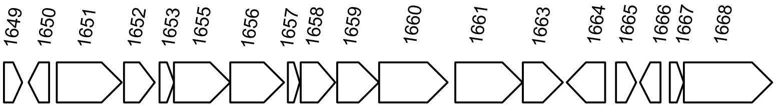

Gene	#AA	Putative function [blast hit species]	% identity	% positive	Accession number	Fold-expression
RVR1649	*138*	hypothetical protein [*Streptomyces atriruber*]	73	79	WP_055568768	1.86
RVR1650	*153*	hypothetical protein [*Streptomyces atriruber*]	70	78	WP_079075063	1.71
RVR1651	*487*	monooxygenase [*Streptomyces atriruber*]	80	87	WP_055568766	1.64
RVR1652	*231*	monooxygenase [*Streptomyces atriruber*]	76	89	WP_055568765	1.27
RVR1653	*109*	cyclase [*Streptomyces* sp. cf124]	83	87	SFO03326	0.80
RVR1655	*423*	β-ketoacyl synthase [*Streptomyces atriruber*]	89	95	WP_055568763	1.60
RVR1656	*403*	chain length determinant [*Streptomyces atriruber*]	88	93	WP_055568762	1.71
RVR1657	*89*	acyl carrier protein [*Streptomyces murayamaensis*]	74	84	AAO65348	1.84
RVR1658	*261*	ketoacyl reductase [*Streptomyces cinnamonensis*]	88	93	CAA77599	1.62
RVR1659	*314*	cyclase [*Streptomyces atriruber*]	83	89	WP_055563829	1.71
RVR1660	*515*	oxidoreductase [*Streptomyces* sp. CNH099]	80	84	WP_051438435	1.57
RVR1661	*506*	oxygenase [*Streptomyces atriruber*]	80	86	WP_055567055	1.57
RVR1663	*301*	thioesterase [*Streptomyces* sp. CNQ-509]	72	78	WP_047019025	0.00
RVR1664	*289*	RNA polymerase sigma factor [*Streptomyces atriruber*]	79	88	WP_055567057	1.11
RVR1665	150	hypothetical protein [*Streptomyces* sp. CNQ-509]	85	91	AKH82283	0.96
RVR1666	*154*	hypothetical protein [*Streptomyces atriruber*]	75	83	WP_055567052	1.17
RVR1667	*108*	ferredoxin [*Streptomyces atriruber*]	78	87	WP_055567051	1.31
RVR1668	*662*	hypothetical protein [*Streptomyces* sp. CNH099]	73	79	WP_027755485	1.30

Cluster 6. T1PKS

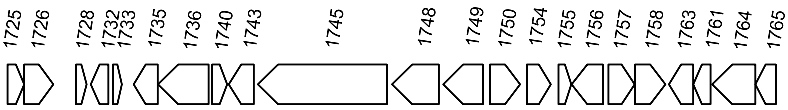

Gene	#AA	Putative function [blast hit species]	% identity	% positive	Accession number	Fold-expression
RVR1725	*224*	hypothetical protein [*Streptomyces* sp. DvalAA-43]	70	80	SCE55747	1.37
RVR1726	*389*	hypothetical protein [*Streptomyces* sp. DvalAA-43]	75	82	SCE55752	0.97
RVR1728	*142*	hypothetical protein [*Streptomyces hygroscopicus*]	59	70	WP_078645663	0.92
RVR1732	*237*	peptidase [*Streptomyces yanglinensis*]	91	94	SEF51752	1.02
RVR1733	*118*	hypothetical protein [*Streptomyces turgidiscabies* Car8]	66	73	ELP66079	0.66
RVR1735	*321*	pirin-like protein [*Streptomyces yeochonensis*]	86	92	WP_037908598	1.60
RVR1736	*674*	hypothetical protein [*Streptomyces* sp. DvalAA-14]	78	82	SCE04969	1.46
RVR1740	*202*	hydrolase [*Streptomyces graminilatus*]	76	85	WP_055534743	1.96
RVR1743	*337*	esterase [*Streptomyces albus subsp. albus*]	61	76	KUJ41291	1.07
RVR1745	*1725*	polyketide synthase [*Streptomyces* sp. CNH189]	53	61	WP_024884684	1.37
RVR1748	*623*	fatty acyl-CoA ligase [*Streptomyces specialis*]	63	71	WP_059013328	1.46
RVR1749	*528*	acyl-CoA carboxylase [*Streptomyces cattleya* NRRL 8057]	73	82	CCB72799	1.54
RVR1750	*402*	transcriptional regulator [*Streptomyces silaceus*]	58	71	WP_079036253	1.73
RVR1754	*320*	fatty acid desaturase [*Streptomyces specialis*]	73	83	WP_059013325	1.56
RVR1755	*171*	gluconokinase [*Streptomyces yanglinensis*]	85	91	SEG57997	0.70
RVR1756	*421*	glucarate dehydratase [*Streptomyces paucisporeus*]	89	93	SHN12345	1.40
RVR1757	*353*	phosphotransferase [*Streptomyces yeochonensis*]	79	84	WP_051950985	1.41
RVR1758	*404*	acyl-CoA dehydrogenase [*Streptomyces yanglinensis*]	95	98	SEG58018	1.01
RVR1763	*327*	dehydrogenase [*Streptomyces paucisporeus*]	84	89	SHN12293	1.71
RVR1761	*231*	tetR family transcriptional regulator [*Streptomyces yanglinensis*]	87	89	SEG58051	1.86
RVR1764	*579*	acyl-CoA synthetase [*Streptomyces alni*]	80	84	SFF32366	1.35
RVR1765	*273*	dehydrogenase [*Streptomyces rubidus*]	88	91	SEO09889	1.50

Cluster 7. T2PKS

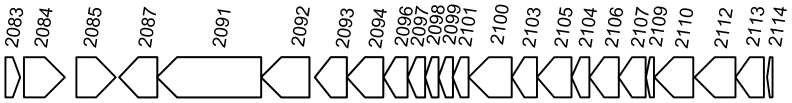

Gene	#AA	Putative function [blast hit species]	% identity	% positive	Accession number	Fold-expression
RVR2083	*161*	hypothetical protein [*Streptomyces rubidus*]	88	94	WP_075016658	1.28
RVR2084	*450*	regulatory protein [*Streptomyces* sp. AA0539]	54	65	WP_019435809	1.19
RVR2085	*431*	dehydrogenase [*Microtetraspora fusca*]	55	65	WP_066943069	1.85
RVR2087	*414*	oxidoreductase [*Streptomyces platensis*]	60	71	WP_085923268	1.08
RVR2091	*1138*	protease [*Actinopolymorpha singaporensis*]	49	61	SDR92660	1.55
RVR2092	*530*	AMP-binding enzyme [*Kutzneria* sp. 744]	66	76	WP_043725653	1.08
RVR2093	*348*	β-ketoacyl synthase [*Streptomyces* sp. AA0539]	58	72	WP_019435811	1.35
RVR2094	*399*	β-ketoacyl synthase [*Kitasatospora setae*]	69	80	WP_014139653	1.44
RVR2096	*263*	acyl carrier protein [*Amycolatopsis vancoresmycina*]	50	58	WP_063749442	1.26
RVR2097	*193*	hypothetical protein [*Kutzneria* sp. 744]	55	72	WP_043725618	1.24
RVR2098	*146*	hypothetical protein [*Kutzneria* sp. 744]	46	63	WP_043725620	1.12
RVR2099	*157*	hypothetical protein [*Streptomyces alni*]	56	64	SFE90037	0.40
RVR2101	*174*	hypothetical protein [*Streptomyces alni*]	51	69	SFE90014	1.30
RVR2100	*471*	multi antimicrobial extrusion protein [*Streptomyces* sp. Tue 6075]	51	65	WP_075264111	1.25
RVR2103	*278*	hypothetical protein [*Kutzneria* sp. 744]	51	66	WP_043725648	1.90
RVR2105	*378*	acyl-CoA dehydrogenase [*Kutzneria* sp. 744]	65	82	EWM18223	1.10
RVR2104	*197*	hypothetical protein [*Streptomyces* sp. NBRC 109706]	63	70	WP_062216619	1.38
RVR2106	*318*	3-oxoacyl-(acyl carrier protein) synthase III [*Amycolatopsis tolypomycina*]	66	75	SEC58367	0.96
RVR2107	*296*	hypothetical protein [*Amycolatopsis tolypomycina*]	54	63	SEC58350	1.32
RVR2109	*87*	phosphopantetheine-binding protein [*Amycolatopsis kentuckyensis*]	74	88	WP_086848245	1.22
RVR2110	*428*	decarboxylase [*Streptomyces* sp. NBRC 109706]	78	86	WP_062216628	1.11
RVR2112	*445*	hypothetical protein [*Streptomyces aidingensis*]	77	84	SFC88550	1.58
RVR2113	*314*	3-oxoacyl-(acyl carrier protein) synthase III [*Amycolatopsis tolypomycina*]	61	75	SEC58234	1.11
RVR2114	*61*	hypothetical protein [*Zancudomyces culisetae*]	62	67	OMH84120	0.00

Cluster 8. Terpene

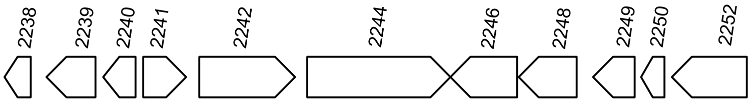

Gene	#AA	Putative function [blast hit species]	% identity	% positive	Accession number	Fold-expression
RVR2238	*138*	membrane protein [*Streptomyces yeochonensis*]	82	90	WP_037906126	1.81
RVR2239	*260*	methyltransferase [*Streptomyces yanglinensis*]	92	95	SEF62604	1.12
RVR2240	*172*	hypothetical protein [*Streptomyces wuyuanensis*]	80	90	SDM15050	0.00
RVR2241	*227*	tetR family transcriptional regulator [*Streptomyces yanglinensis*]	92	96	SEF62539	0.90
RVR2242	*507*	FAD dependent oxidoreductase [*Streptomyces yanglinensis*]	77	82	SEF62513	0.98
RVR2244	*758*	germacradienol/geosmin synthase [*Streptomyces yanglinensis*]	84	89	SEF62480	0.91
RVR2246	*354*	hypothetical protein [*Streptomyces yanglinensis*]	67	72	SEF62448	0.82
RVR2248	*306*	methylenetetrahydrofolate reductase [*Streptomyces yanglinensis*]	95	98	SEF62421	1.02
RVR2249	*221*	thiamine-phosphate pyrophosphorylase [*Streptomyces* sp. LamerLS-31b]	78	85	SCF83044	1.03
RVR2250	*124*	transcriptional regulator [*Streptomyces guanduensis*]	91	96	SDN70938	0.49
RVR2252	*397*	ferredoxin reductase [*Streptomyces yanglinensis*]	88	94	SEF62329	0.44

Cluster 9. T1PKS

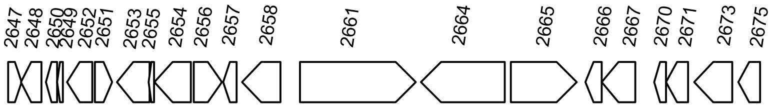

Gene	#AA	Putative function [blast hit species]	% identity	% positive	Accession number	Fold-expression
RVR2647	*165*	cytidyltransferase [*Streptomyces yanglinensis*]	89	90	SEF55187	0.79
RVR2648	*254*	phosphatidyltransferase [*Streptomyces yanglinensis*]	83	88	SEF55144	0.36
RVR2650	*139*	hypothetical protein [*Streptomyces vitaminophilus*]	84	92	WP_018386448	0.76
RVR2649	*72*	hypothetical protein [*Streptomyces albus*]	87	95	WP_030543475	0.15
RVR2652	*312*	aldo/keto reductase [*Streptacidiphilus albus*]	74	85	WP_034090702	0.96
RVR2651	*214*	TetR family transcriptional regulator [*Nocardia paucivorans*]	65	77	WP_085995128	1.00
RVR2653	*406*	β-lactamase [*Streptomyces yanglinensis*]	79	84	SEF55101	1.29
RVR2655	*64*	hypothetical protein [*Streptomyces yanglinensis*]	95	98	SEF55074	0.03
RVR2654	*449*	transporter [*Streptomyces yanglinensis*]	85	90	SEF55049	1.84
RVR2656	*347*	oxidoreductase [*Streptomyces yanglinensis*]	86	91	SEF55024	1.04
RVR2657	*164*	acetyltransferase [*Streptomyces yanglinensis*]	84	90	SEF54778	1.04
RVR2658	*476*	decarboxylase [*Streptomyces yanglinensis*]	71	79	SEF54639	1.11
RVR2661	*1431*	polyketide synthase [*Streptomyces yanglinensis*]	64	72	SEF54608	0.93
RVR2664	*1039*	transcriptional regulator [*Streptomyces yanglinensis*]	70	78	SEF54445	1.01
RVR2665	*813*	transcriptional regulator [*Streptomyces yanglinensis*]	66	75	SEF54409	1.48
RVR2666	*202*	hypothetical protein [*Streptomyces* sp. MP131-18]	44	53	ONK15509	1.00
RVR2667	*415*	hypothetical protein [*Streptomyces yanglinensis*]	81	88	SEF54346	1.34
RVR2670	*143*	hypothetical protein [*Streptomyces yanglinensis*]	79	84	SEF54215	2.74
RVR2671	*274*	oxidoreductase [*Streptomyces guanduensis*]	84	91	SDM79912	1.01
RVR2673	*469*	hypothetical protein [*Streptomyces yanglinensis*]	73	83	SEF53760	0.91
RVR2675	*271*	hypothetical protein [*Streptomyces yanglinensis*]	77	85	SEG93265	0.64

Cluster 10. T1PKS

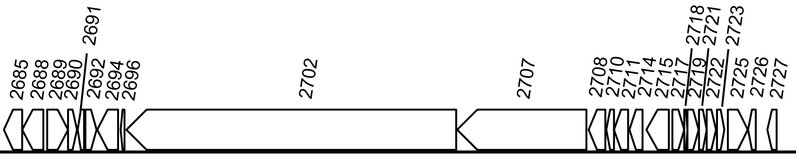

Gene	#AA	Putative function [blast hit species]	% identity	% positive	Accession number	Fold-expression
RVR2685	*451*	gluconate permease [*Streptomyces* sp. NRRL F-5630]	75	86	WP_030994718	1.13
RVR2688	*512*	hypothetical protein [*Streptomyces sulphureus*]	69	76	WP_019544709	0.92
RVR2689	*512*	hypothetical protein [*Streptomyces* sp. FXJ7.023]	57	63	WP_037770801	0.67
RVR2690	*214*	aldolase [*Streptomyces* sp. Root55]	69	76	WP_056789882	1.47
RVR2691	*159*	phenylacetic acid degradation protein [*Streptomyces atratus*]	74	85	WP_037697982	1.41
RVR2692	*272*	hydrolase [*Streptomyces violaceoruber*]	71	82	WP_030947178	2.45
RVR2694	*514*	acyl-CoA synthetase [*Amycolatopsis sacchari*]	75	85	SFJ41708	3.19
RVR2696	*93*	hypothetical protein [*Streptomyces prunicolor*]	96	97	WP_019060185	4.88
RVR2702	*8084*	polyketide synthase [*Streptomyces lasaliensis*]	62	73	BAQ03814	2.64
RVR2707	*3172*	polyketide synthase [*Micromonospora echinospora*]	53	65	SCF42107	2.76
RVR2708	*405*	cytochrome P450 [*Streptomyces prunicolor*]	95	98	WP_019065465	3.36
RVR2710	*183*	hypothetical protein [*Mycobacterium riyadhense*]	59	76	WP_085251430	3.27
RVR2711	*346*	hypothetical protein [*Mycobacterium sp. TBL 1200985*]	46	59	WP_085324407	3.07
RVR2714	*319*	hypothetical protein [*Streptomyces prunicolor*]	93	98	WP_019065480	3.07
RVR2715	*545*	AMP-dependent synthetase [*Streptomyces prunicolor*]	89	93	WP_043259180	3.35
RVR2717	*290*	hypothetical protein [*Streptomyces prunicolor*]	93	96	WP_019065482	2.27
RVR2718	*79*	hypothetical protein [*Streptomyces prunicolor*]	97	100	WP_019065483	1.82
RVR2719	*280*	hypothetical protein [*Streptomyces prunicolor*]	87	93	WP_019065484	2.66
RVR2721	*188*	hypothetical protein, partial [*Mycobacterium riyadhense*]	59	76	WP_085251430	2.57
RVR2722	*226*	hypothetical protein [*Streptomyces prunicolor*]	92	96	WP_052347109	2.46
RVR2723	*184*	isoprenylcysteine carboxylmethyltransferase family protein [*Streptomyces prunicolor*]	83	91	WP_019062026	3.03
RVR2725	*492*	major facilitator superfamily transporter [*Streptomyces prunicolor*]	87	93	WP_019062027	2.28
RVR2726	*188*	transcriptional regulator, LuxR family [*Streptomyces prunicolor*]	83	89	WP_019062028	1.66
RVR2727	*228*	transcriptional regulator, LuxR family [*Streptomyces prunicolor*]	84	89	WP_019062029	2.21

Cluster 11. Fatty acid

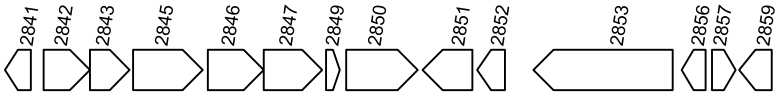

Gene	#AA	Putative function [blast hit species]	% identity	% positive	Accession number	Fold-expression
RVR2841	*153*	MerR family transcriptional regulator [*Streptomyces yanglinensis*]	84	90	SEF48860	1.28
RVR2842	*273*	β-lactamase [*Streptomyces yanglinensis*]	85	89	SEF48829	0.77
RVR2843	*235*	hypothetical protein [*Streptomyces yanglinensis*]	79	82	SEF48794	1.36
RVR2845	*410*	hypothetical protein [*Streptomyces yanglinensis*]	97	97	SEF48760	1.19
RVR2846	*331*	acyl carrier protein S-malonyltransferase [*Streptomyces yeochonensis*]	80	87	WP_051950744	0.59
RVR2847	*344*	3-oxoacyl-acyl carrier protein synthase III [*Streptomyces yanglinensis*]	94	98	SEF48697	0.68
RVR2849	*81*	acyl carrier protein [*Streptomyces yanglinensis*]	97	100	SEF48666	0.67
RVR2850	*422*	3-oxoacyl-(acyl carrier protein) synthase [*Streptomyces yanglinensis*]	94	97	SEF48633	0.88
RVR2851	*293*	dehydrogenase [*Streptomyces yanglinensis*]	79	86	SEF48604	1.23
RVR2852	*164*	hypothetical protein n [*Streptomyces yanglinensis*]	97	98	SEF48574	1.46
RVR2853	*820*	β-glucosidase [*Streptomyces yanglinensis*]	82	87	SEF48505	0.85
RVR2856	*142*	organic hydroperoxide resistance protein [*Streptomyces scopuliridis*]	87	94	WP_030352557	1.84
RVR2857	*139*	MarR family transcriptional regulator [*Streptomyces yanglinensis*]	81	86	SEF48429	1.67
RVR2859	*192*	hypothetical protein [*Streptomyces yanglinensis*]	79	85	SEF48360	0.62

Cluster 12. T2PKS

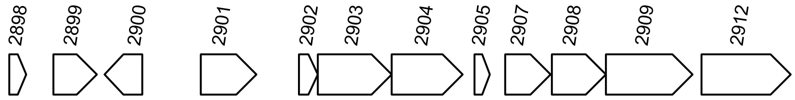

Gene	#AA	Putative function [blast hit species]	% identity	% positive	Accession number	Fold-expression
RVR2898	*97*	hypothetical protein [*Streptomyces*]	70	75	WP_030377316	1.25
RVR2899	*250*	hydrolase [*Streptomyces antioxidans*]	75	85	WP_046088292	1.44
RVR2900	*218*	transcriptional regulatory protein [*Streptomyces bicolor*]	60	77	WP_031478691	1.00
RVR2901	*322*	hypothetical protein [*Streptomyces* sp. NBRC 109706]	57	64	WP_062214984	1.04
RVR2902	*108*	cyclase [*Streptomyces* sp. MJM1172]	90	92	WP_073775778	1.21
RVR2903	*426*	β-ketoacyl synthase [*Streptomyces* sp. MJM1172]	91	95	WP_073775776	1.10
RVR2904	*409*	chain length determinant [*Streptomyces* sp.]	88	91	APD71655	1.03
RVR2905	*89*	acyl carrier protein [*Streptomyces nodosus*]	80	94	AJE43677	0.69
RVR2907	*261*	ketoreductase [*Streptomyces* sp. MJM1172]	88	93	WP_073775770	1.21
RVR2908	*311*	aromatase [*Streptomyces* sp. MJM1172]	82	89	WP_073775768	1.11
RVR2909	*501*	oxygenase [*Streptomyces* sp. MJM1172]	75	80	WP_073775766	1.00
RVR2912	*514*	oxygenase [*Streptomyces* sp. MJM1172]	79	84	WP_073775764	1.25

Cluster 13. T2PKS

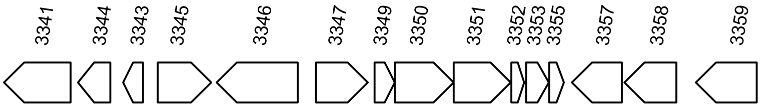

Gene	#AA	Putative function [blast hit species]	% identity	% positive	Accession number	Fold-expression
RVR3341	*491*	sugar hydrolase [*Streptomyces yanglinensis*]	82	89	SEG79477	1.12
RVR3344	*238*	secreted protein [*Streptomyces yanglinensis*]	74	85	SEG79471	1.15
RVR3343	*145*	hypothetical protein [*Streptomyces* sp. SirexAA-E]	51	65	WP_014044486	1.13
RVR3345	*394*	hypothetical protein [*Streptomyces yanglinensis*]	81	86	SEG79449	1.21
RVR3346	*593*	polyketide hydroxylase [*Streptomyces yanglinensis*]	81	86	SEG79441	1.23
RVR3347	*382*	WhiE I protein [*Streptomyces yanglinensis*]	79	84	SEG79435	1.04
RVR3349	*147*	WhiE II protein [*Streptomyces guanduensis*]	81	89	SDO06260	1.16
RVR3350	*434*	β-ketoacyl synthase [*Streptomyces yanglinensis*]	90	95	SEG79422	1.24
RVR3351	*419*	chain length determinant [*Streptomyces yanglinensis*]	89	93	SEG79415	0.97
RVR3352	*94*	acyl carrier protein [*Streptomyces yanglinensis*]	73	83	SEG79409	0.57
RVR3353	*168*	cyclase [*Streptomyces yanglinensis*]	87	93	SEG79402	1.03
RVR3355	*108*	cyclase [*Streptomyces yanglinensis*]	83	91	SEG79396	1.23
RVR3357	*366*	*O*-methyltransferase [*Streptomyces yeochonensis*]	82	89	WP_037907854	1.03
RVR3358	*380*	secreted protein [*Streptomyces guanduensis*]	68	76	SDO06055	0.83
RVR3359	*447*	sensor kinase [*Streptomyces yanglinensis*]	93	95	SEG79368	1.40

Cluster 14. T3PKS

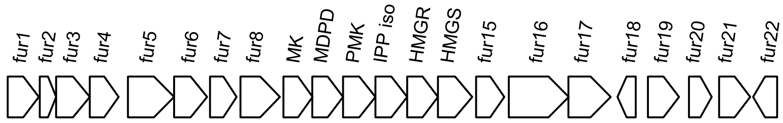

Gene	#AA	Putative function [blast hit species]	% identity	% positive	Accession number	Fold-expression
RVR4160 (*fur1*)	*356*	polyketide synthase [*Streptomyces antibioticus*]	89	95	BAB91443	0.69
RVR4159 (*fur2*)	*188*	monooxygenase [*Streptomyces* sp. NRRL S-37]	86	93	WP_030857819	0.50
RVR4158 (*fur3*)	*387*	aminotransferase [*Streptomyces yokosukanensis*]	88	93	WP_067123095	0.55
RVR4156 (*fur4*)	*331*	methyltransferase [*Streptomyces* sp. KO-3988]	86	91	BAE78972	0.60
RVR4155 (*fur5*)	*528*	fatty acyl-CoA ligase [*Streptomyces* sp. KO-3988]	87	93	BAE78973	0.79
RVR4154 (*fur6*)	*378*	methyltransferase [*Streptomyces* sp. KO-3988]	90	94	BAE78974	0.57
RVR4153 (*fur7*)	*305*	prenyltransferase [*Streptomyces* sp. KO-3988]	84	91	BAE78975	0.65
RVR4151 (*fur8*)	*455*	P450 [*Streptomyces* sp. KO-3988]	78	86	BAE78976	0.59
RVR4149 (*fur9*)	*333*	mevalonate kinase [*Streptomyces* sp. KO-3988]	78	84	BAE78977	0.87
RVR4148 (*fur10*)	*351*	diphosphomevalonate decarboxylase [*Streptomyces* sp. NRRL S-37]	83	88	WP_051767179	0.73
RVR4147 (*fur11*)	*371*	phosphomevalonate kinase [*Streptomyces* sp. KO-3988]	80	84	BAE78979	1.00
RVR4146 (*fur12*)	*363*	type II isopentenyl diphosphate isomerase [*Streptomyces* sp. KO-3988]	86	91	BAE78980	0.92
RVR4144 (*fur13*)	*352*	3-hydroxy-3-methylglutaryl coenzyme A reductase [*Streptomyces* sp. CL190]	89	95	BAA70975	0.63
RVR4143 (*fur14*)	*391*	3-hydroxy-3-methylglutaryl-CoA synthase [*Streptomyces* sp. KO-3988]	88	94	BAE78982	0.75
RVR4142 (*fur15*)	*324*	acetoacetyl-CoA synthase [*Streptomyces* sp. KO-3988]	87	92	BAE78983	0.74
RVR4141 (*fur16*)	*686*	hypothetical protein [*Streptomyces* sp. KO-3988]	78	84	BAE78984	0.95
RVR4139 (*fur17*)	*481*	carboxymuconate cycloisomerase [*Streptomyces* sp. KO-3988]	88	91	BAE78985	0.57
RVR4137 (*fur18*)	*209*	ovmZ homologue [*Streptomyces* sp. KO-3988]	67	78	BAE78986	0.79
RVR4135 (*fur19*)	*355*	geranyl diphosphate synthase [*Streptomyces* sp. KO-3988]	81	88	BAE78987	1.03
RVR4133 (*fur20*)	*259*	prenyl diphosphate synthase [*Streptomyces* sp. KO-3988]	60	72	BAE78988	0.97
RVR4132 (*fur21*)	*356*	methyltransferase [*Streptomyces* sp. KO-3988]	81	89	BAE78989	0.83
RVR4131 (*fur22*)	*273*	transcriptional regulator [*Streptomyces* sp. KO-3988]	83	89	BAE78990	0.96

Cluster 15. Prenylindole[2]

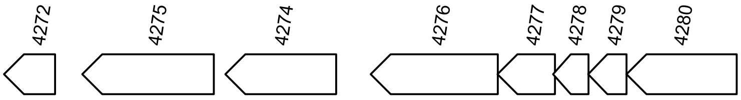

Gene	#AA	Putative function [blast hit species]	% identity	% positive	Accession number	Fold-expression
RVR4272	*178*	hypothetical protein [*Streptomyces roseochromogenus*]	70	85	WP_031223882	2.02
RVR4275	*457*	tryptophanse [*Streptomyces yanglinensis*]	92	96	SEG88414	1.35
RVR4274 (*iptA*)	*385*	6-dimethylallyl-L-Trp synthase [*Streptomyces yanglinensis*]	65	74	SEG88418	1.82
RVR4276	*441*	cytochrome P450 [*Streptomyces yanglinensis*]	87	93	SEG88422	0.74
RVR4277	*200*	ATP/GTP-binding protein [*Streptomyces yanglinensis*]	92	94	SEG88425	0.58
RVR4278	*123*	hypothetical protein [*Streptomyces yanglinensis*]	86	93	SEG88429	0.59
RVR4279	*133*	hypothetical protein [*Streptomyces yanglinensis*]	92	94	SEG88438	0.63
RVR4280	*382*	sensor histidine kinase [*Streptomyces yanglinensis*]	87	91	SEG88443	0.78

Cluster 16. T1PKS

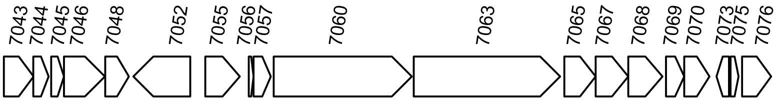

Gene	#AA	Putative function [blast hit species]	% identity	% positive	Accession number	Fold-expression
RVR7043	*517*	amidohydrolase [*Streptomyces griseoflavus*]	75	86	WP_004924025	0.15
RVR7044	*267*	dihydroxybenzoate dehydrogenase [*Streptomyces scabiei* 87.22]	63	75	YP_003486941	0.11
RVR7045	*223*	isochorismatase [*Streptomyces antibioticus*]	70	78	AFB35627	0.23
RVR7046	*715*	chorismate binding enzyme [*Streptomyces antibioticus*]	60	71	AFB35626	0.27
RVR7048	*413*	phospho-2-dehydro-3-deoxyheptonate aldolase [*Streptomyces griseoflavus*]	68	76	WP_004924033	0.29
RVR7052	*993*	transcriptional regulator, LuxR family [*Streptomyces* sp. Tu 6176]	48	63	WP_018564652	0.28
RVR7055	*602*	transcriptional regulator [*Streptomyces* sp. PTY087I2]	47	62	OCC10617	0.58
RVR7056	*87*	phosphopantetheine binding protein [*Kitasatospora* sp. MBT63]	82	90	WP_082527277	0.06
RVR7057	*305*	thioesterase [*Streptomyces rhizosphaericus*]	68	74	WP_086881632	0.08
RVR7060	*2404*	polyketide synthase [*Nocardia* sp. BMG111209]	50	62	WP_019927420	0.16
RVR7063	*2545*	polyketide synthase [*Streptomyces* sp. PsTaAH-124]	76	83	WP_018564631	0.20
RVR7065	*548*	monooxygenase [*Streptomyces* sp. PsTaAH-124]	83	90	WP_018564630	0.22
RVR7067	*568*	acyl-CoA dehydrogenase [*Streptomyces* sp. PsTaAH-124]	75	82	WP_018564629	0.19
RVR7068	*597*	acyl-CoA dehydrogenase [*Streptomyces* sp. e14]	80	87	WP_009191833	0.17
RVR7069	*318*	phosphopantetheinyl transferase [*Streptomyces* sp. Amel2xE9]	54	61	WP_019983399	0.40
RVR7070	*440*	amine oxidase [*Streptomyces* sp. Amel2xE9]	78	88	WP_019983400	0.28
RVR7073	*216*	transcriptional regulator [*Streptomyces agglomeratus*]	70	80	WP_069928026	0.85
RVR7075	*140*	hypothetical protein [*Streptacidiphilus carbonis*]	76	87	WP_042402256	0.98
RVR7076	*503*	transmembrane efflux protein [*Streptomyces griseoflavus*]	70	82	WP_084828113	1.29

Cluster 17. Squalene hopene

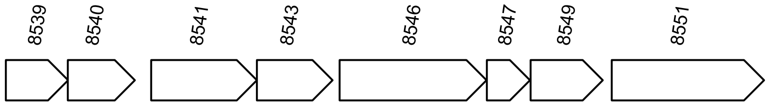

Gene	#AA	Putative function [blast hit species]	% identity	% positive	Accession number	Fold-expression
RVR8539	*295*	squalene synthase [*Streptomyces yanglinensis*]	92	94	SEG17405	0.81
RVR8540	*318*	squalene synthase [*Streptomyces yanglinensis*]	93	95	SEG17435	0.95
RVR8541	*502*	phytoene dehydrogenase [*Streptomyces yanglinensis*]	86	90	SEG17457	0.80
RVR8543	*359*	polyprenyl diphosphate synthase [*Streptomyces yanglinensis*]	97	99	SEG17483	0.73
RVR8546	*697*	squalene-hopene cyclase [*Streptomyces yanglinensis*]	91	94	SEG17515	0.98
RVR8547	*205*	hypothetical protein [*Streptomyces yanglinensis*]	92	94	SEG17540	0.98
RVR8549	*342*	hopanoid biosynthesis associated radical SAM protein [*Streptomyces yanglinensis*]	97	100	SEG17570	0.85
RVR8551	*722*	1-deoxy-D-xylulose-5-phosphate synthase [*Streptomyces yanglinensis*]	86	89	SEG17595	0.81

Cluster 18. T1PKS (RM-A)[3-5]

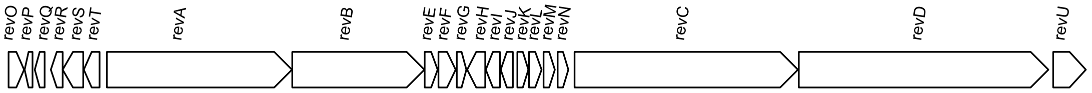

Gene	#AA	Putative function [blast hit species]	% identity	% positive	Accession number	Fold-expression
RVR8611 (*revO*)	443	transporter [*Streptomyces* sp. HGB0020]	83	91	EPD63749	1.12
RVR8613 (*revP*)	224	transcriptional regulator, GntR family [*Streptomyces* sp. PAMC 26508]	72	84	AGJ58777	0.79
RVR8614 (*revQ*)	277	transcriptional regulator, SARP family [*Streptomyces violaceusniger* Tu 4113]	82	91	AEM85964	1.36
RVR8616 (*revR*)	333	3-oxoacyl-[acyl-carrier-protein] synthase [*Kitasatospora setae* KM-6054]	66	79	BAJ32310	4.07
RVR8617 (*revS*)	579	medium chain fatty acyl-CoA ligase [*Kitasatospora setae* KM-6054]	65	76	BAJ32311	5.35
RVR8618 (*revT*)	443	(*E*)-2-hexenoyl-CoA reductase carboxylase [*Kitasatospora setae* KM-6054]	72	85	BAJ32312	6.30
RVR8622 (*revA*)	5210	polyketide synthase [*Streptomyces violaceusniger* Tu 4113]	78	86	AEM85978	4.43
RVR8628 (*revB*)	3724	polyketide synthase [*Streptomyces violaceusniger* Tu 4113]	79	86	AEM85977	4.87
RVR8630 (*revE*)	*367*	dehydrogenase [*Streptomyces violaceusniger* Tu 4113]	78	87	AEM85976	4.90
RVR8631 (*revF*)	*483*	aldehyde dehydrogenase [*Streptomyces violaceusniger* Tu 4113]	81	91	AEM85975	6.08
RVR8633 (*revG*)	*273*	dihydroxyketone synthase [*Streptomyces violaceusniger* Tu 4113]	90	94	AEM85974	6.89
RVR8634 (*revH*)	*507*	FAD dependent oxidoreductase [*Streptomyces violaceusniger* Tu 4113]	80	88	AEM85973	6.66
RVR8636 (*revI*)	*397*	reveromycin T hydroxylase [*Streptomyces* sp. AA4]	64	78	EFL09915	8.79
RVR8637 (*revJ*)	*336*	spiroacetal synthase [*Streptomyces violaceusniger* Tu 4113]	77	88	AEM85972	5.67
RVR8638 (*revK*)	*314*	transferase [*Streptomyces* sp. PRh5]	41	59	EXU64038	7.60
RVR8649 (*revL*)	*357*	hypothetical protein [*Streptomyces* sp. PRh5]	63	76	EXU64039	5.35
RVR8640 (*revM*)	*320*	oxidoreductase [*Actinoplanes missouriensis* 431]	74	83	BAL93224	4.57
RVR8641 (*revN*)	*305*	esterase [*Streptomyces violaceusniger* Tu 4113]	88	95	AEM85971	2.66
RVR8651 (*revC*)	*6284*	polyketide synthase [*Streptomyces violaceusniger* Tu 4113]	81	87	AEM85970	4.02
RVR8659 (*revD*)	*7030*	polyketide synthase [*Streptomyces violaceusniger* Tu 4113]	80	86	AEM85968	3.52
RVR8661 (*revU*)	*923*	transcriptional regulator, LuxR family [*Streptomyces violaceusniger* Tu 4113]	78	85	AEM85967	2.78

Cluster 19. Terpene

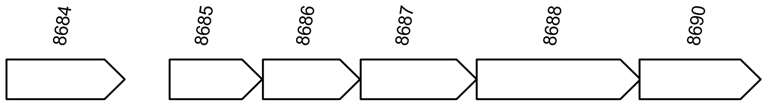

Gene	#AA	Putative function [blast hit species]	% identity	% positive	Accession number	Fold-expression
RVR8684	*413*	cytochrome P450 [*Streptosporangium roseum* DSM 43021]	56	69	YP_003338846	1.65
RVR8685	*323*	aminotransferase [*Streptomyces griseoaurantiacus*]	60	69	WP_006138668	1.64
RVR8686	*340*	polyprenyl synthetase [*Streptomyces griseoaurantiacus*]	61	72	WP_006138667	1.71
RVR8687	*406*	prenyltransferase [*Actinobacteria bacterium* OK074]	59	68	KPI08360	1.52
RVR8688	*571*	terpene synthase [*Saccharopolyspora erythraea* NRRL 2338]	71	78	YP_001106842	1.83
RVR8690	*423*	FAD-dependent monooxygenase [*Saccharopolyspora spinosa*]	65	73	WP_010312129	1.52

Cluster 20. T1PKS

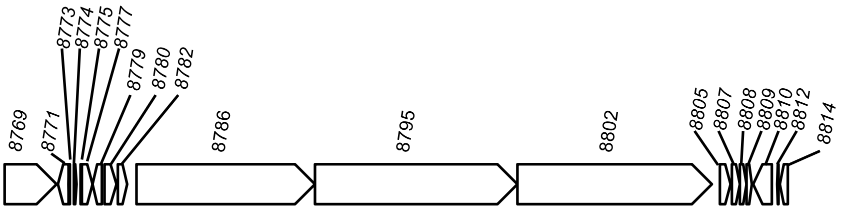

Gene	#AA	Putative function [blast hit species]	% identity	% positive	Accession number	Fold-expression
RVR8769	*2112*	polyketide synthase [*Streptomyces rapamycinicus* NRRL 5491]	90	93	WP_020872759	1.31
RVR8771	*423*	cytochrome P450 [*Streptomyces rapamycinicus* NRRL 5491]	93	96	YP_008794234	0.93
RVR8773	*66*	ferredoxin [*Streptomyces rapamycinicus* NRRL 5491]	87	95	YP_008794237	0.66
RVR8774	*109*	hypothetical protein [*Streptomyces rapamycinicus* NRRL 5491]	76	80	YP_008794236	1.11
RVR8775	*71*	ferredoxin [*Streptomyces rapamycinicus* NRRL 5491]	87	93	YP_008794237	1.00
RVR8777	*435*	cytochrome P450 [*Streptomyces rapamycinicus* NRRL 5491]	92	96	YP_008794238	1.12
RVR8779	*371*	uroporphyrinogen decarboxylase [*Streptomyces rapamycinicus* NRRL 5491]	95	97	YP_008794239	1.25
RVR8780	*499*	aldehyde dehydrogenase [*Streptomyces rapamycinicus* NRRL 5491]	95	97	YP_008794240	1.18
RVR8782	*423*	cytochrome P450 [*Streptomyces rapamycinicus* NRRL 5491]	89	94	YP_008794241	1.25
RVR8786	*7358*	polyketide synthase [*Streptomyces rapamycinicus* NRRL 5491]	87	91	YP_008794245	1.21
RVR8795	*8353*	polyketide synthase [*Streptomyces rapamycinicus* NRRL 5491]	79	85	YP_008794245	1.18
RVR8802	*8021*	polyketide synthase [*Micromonospora* sp. L5]	50	61	YP_004085507	1.23
RVR8805	*435*	extracellular solute-binding protein [*Streptomyces* sp. NRRL F-5123]	76	84	WP_052397960	1.36
RVR8807	*321*	multiple sugar ABC transporter permease [*Streptomyces paucisporeus*]	80	89	WP_073498310	1.31
RVR8808	*296*	multiple sugar ABC transporter permease [*Streptomyces paucisporeus*]	87	94	WP_079189746	1.47
RVR8809	*218*	hypothetical protein [*Streptomyces paucisporeus*]	61	71	WP_073498308	0.88
RVR8810	*759*	secreted glycosyl hydrolase [*Streptomyces paucisporeus*]	79	88	WP_073501039	1.69
RVR8812	*88*	hypothetical protein [*Streptomyces* sp. DvalAA-14]	89	93	SCE41692	1.07
RVR8814	*361*	hypothetical protein [*Streptomyces* sp. DvalAA-14]	83	89	SCE51818	1.06

Cluster 21. T2PKS

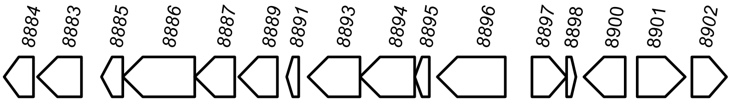

Gene	#AA	Putative function [blast hit species]	% identity	% positive	Accession number	Fold-expression
RVR8884	*225*	monooxygenase [*Streptomyces yanglinensis*]	84	92	SEG87481	1.52
RVR8883	*336*	*O*-methyltransferase [*Streptomyces yanglinensis*]	85	88	SEG87477	1.85
RVR8885	*167*	hypothetical protein [*Streptomyces yanglinensis*]	80	86	SEG87471	1.82
RVR8886	*556*	oxygenase [*Streptomyces yanglinensis*]	84	88	SEG87465	2.03
RVR8887	*314*	aromatase [*Streptomyces* sp. WP 4669]	83	90	SEG87461	1.57
RVR8889	*304*	ketoreductase [*Streptomyces yanglinensis*]	88	92	SEG87456	1.75
RVR8891	*91*	acyl carrier protein [*Streptomyces yanglinensis*]	89	96	SEG87450	1.55
RVR8893	*406*	chain length determinant [*Streptomyces yanglinensis*]	92	95	SEG87444	1.64
RVR8894	*423*	β-ketoacyl synthase [*Streptomyces yanglinensis*]	94	96	SEG87439	1.71
RVR8895	*109*	cyclase [*Streptomyces yanglinensis*]	90	93	SEG87435	1.33
RVR8896	*534*	oxygenase [*Streptomyces yanglinensis*]	81	84	SEG87429	1.46
RVR8897	*260*	hypothetical protein [*Streptomyces yanglinensis*]	75	81	SEG87422	1.82
RVR8898	*76*	hypothetical protein [*Streptomyces* sp. AW19M42]	86	93	WP_024491206	1.00
RVR8900	*320*	dehydrogenase [*Streptomyces* sp. AcH 505]	81	86	WP_041993210	1.00
RVR8901	*368*	pyridine nucleotide-disulfide oxidoreductase [*Streptomyces yanglinensis*]	85	90	SEG87410	1.62
RVR8902	*267*	dehydrogenase [*Streptomyces yanglinensis*]	92	95	SEG87404	1.43

Cluster 22. T3PKS

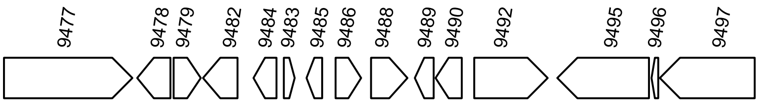

Gene	#AA	Putative function [blast hit species]	% identity	% positive	Accession number	Fold-expression
RVR9477	*1393*	hypothetical protein [*Streptomyces yanglinensis*]	73	81	SEG82223	1.36
RVR9478	*362*	alcohol dehydrogenase [*Streptomyces rimosus*]	83	89	WP_003979245	1.73
RVR9479	*295*	LysR family transcriptional regulator [*Streptomyces rimosus*]	77	84	WP_003979246	1.16
RVR9482	*375*	dehydrogenase [*Streptomyces yanglinensis*]	84	91	SEG93157	1.29
RVR9484	*249*	hypothetical protein [*Streptomyces ghanaensis*]	68	80	WP_004993778	1.18
RVR9483	*120*	hypothetical protein [*Streptomyces* sp. yr375]	77	85	SER12559	0.00
RVR9485	*169*	hypothetical protein [*Streptomyces guanduensis*]	58	67	SDM82724	1.25
RVR9486	*276*	hypothetical protein [*Streptomyces guanduensis*]	75	85	SDM82755	1.05
RVR9488	*393*	polyketide synthase [*Streptomyces guanduensis*]	79	85	SDM82782	0.94
RVR9489	*204*	integral membrane protein [*Streptomyces* sp. URHA0041]	75	82	WP_033178380	0.46
RVR9490	*285*	hypothetical protein [*Streptomyces* sp. DvalAA-14]	62	70	SCE07136	0.47
RVR9492	*794*	protein phosphatase [*Streptomyces* niveus]	49	61	WP_079128081	1.34
RVR9495	*993*	transcriptional regulator, LuxR family [*Streptomyces alni*]	90	93	SFF66258	1.18
RVR9496	*72*	hypothetical protein [*Streptomyces alni*]	83	82	SFF66268	0.00
RVR9497	*1028*	lanthionine synthetase [*Streptomyces malaysiense*]	78	82	WP_053055598	1.36

Cluster 23. T1PKS/NRPS

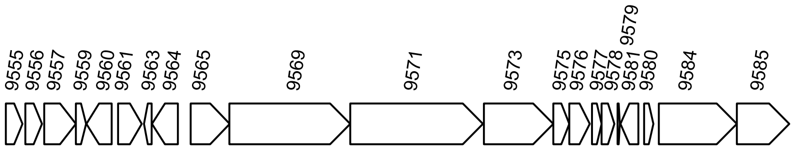

Gene	#AA	Putative function [blast hit species]	% identity	% positive	Accession number	Fold- expression
RVR9555	*270*	short chain dehydrogenase/reductase [*Rhodococcus wratislaviensis*]	48	64	WP_005563150	0.38
RVR9556	*269*	enoyl-coA hydratase [*Bradyrhizobium* sp. LTSPM299]	54	66	WP_083215819	0.47
RVR9557	*509*	acyl-CoA synthetase [*Cupriavidus necator*]	46	62	WP_078199589	0.43
RVR9559	*165*	hypothetical protein [*Bradyrhizobium erythrophlei*]	40	55	SEE25997	0.75
RVR9560	*406*	transposase [*Nocardiopsis* sp. CNR-923]	85	90	WP_083678388	0.77
RVR9561	*380*	thiolase [*Bacillus dakarensis*]	59	71	WP_077213191	0.65
RVR9563	*126*	hypothetical protein [*Acidimicrobium ferrooxidans* DSM 10331]	37	55	YP_003110203	1.06
RVR9564	*417*	cytochrome P450 [*Cryptosporangium aurantiacum*]	45	63	WP_073256405	1.32
RVR9565	627	carbamoyltransferase [*Burkholderia glumae* BGR1]	50	61	YP_002907570	0.96
RVR9569	1950	nonribosomal peptide synthetase [*Scytonema hofmannii* PCC 7110]	34	50	KYC42747	0.91
RVR9571	2145	polyketide synthase [*Paenibacillus borealis*]	36	52	OMD47781	1.00
RVR9573	*1116*	nonribosomal peptide synthetase [*Streptomyces collinus*]	35	47	WP_020937617	1.03
RVR9575	*260*	thioesterase [*Cylindrospermum stagnale* PCC 7417]	36	51	YP_007150861	0.98
RVR9576	*332*	polyprenyl diphosphate synthase [*Streptomyces* sp. BoleA5]	60	73	WP_018558806	1.02
RVR9577	*155*	hypothetical protein [*Salinispora arenicola*]	38	57	WP_018806283	0.92
RVR9578	*209*	hydrolase [*Salinispora arenicola*]	48	57	WP_029027497	0.00
RVR9579	*39*	hypothetical protein [*Streptomyces* sp. NTK 937]	79	88	WP_051674029	0.00
RVR9581	*289*	short chain dehydrogenase [*Streptomyces* sp. DvalAA-14]	81	88	SCE29449	1.40
RVR9580	*146*	hypothetical protein [*Streptomyces collinus* Tu 365]	68	76	YP_008384318	0.89
RVR9584	*1257*	two-component system sensor kinase [*Streptomyces mobaraensis*]	74	83	WP_004940803	0.86
RVR9585	*848*	protein phosphatase [*Streptomyces mobaraensis*]	66	77	WP_004940802	1.04

Cluster 24. PKS/NRPS

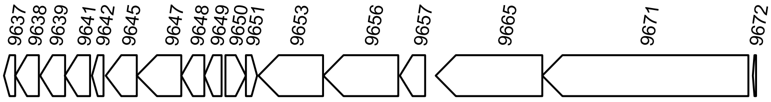

Gene	#AA	Putative function [blast hit species]	% identity	% positive	Accession number	Fold-expression
RVR9637	*266*	acetylglutamate kinase [*Synechococcus* sp. PCC 7002]	43	63	YP_001735749	0.81
RVR9638	*564*	nonribosomal peptide synthetase [*Pseudomonas syringae*]	33	51	WP_016568293	0.70
RVR9639	*593*	ABC transporter ATP-binding protein [*Amycolatopsis mediterranei* U32]	48	60	YP_003767702	0.92
RVR9641	*600*	ABC transporter ATP-binding protein [*Nocardiopsis* sp. CNS639]	43	59	WP_019607514	0.71
RVR9642	*278*	short chain dehydrogenase [*Streptosporangium canum*]	58	71	SFJ03028	0.73
RVR9645	*739*	amidase [*Methylobacter marinus*]	40	53	WP_020158459	0.69
RVR9647	*1052*	nonribosomal peptide synthetase [*Streptomyces* sp. NRRL S-1868]	37	49	WP_051856098	0.67
RVR9648	*549*	nonribosomal peptide synthetase [*Myxococcus hansupus*]	33	48	WP_049872272	0.75
RVR9649	*415*	monooxygenase [*Cystobacter fuscus*]	51	65	WP_002628882	0.83
RVR9650	*493*	nonribosomal peptide synthetase [*Corallococcus coralloides* DSM 2259]	36	50	YP_005368577	0.71
RVR9651	*261*	thioesterase [*Pseudanabaena biceps*]	44	57	WP_009628113	0.80
RVR9653	*1534*	polyketide synthase [*Fischerella* sp. PCC 9431]	41	60	WP_026723923	0.94
RVR9656	*1776*	nonribosomal peptide synthetase [*Clostridium termitidis*]	33	51	WP_004623654	0.96
RVR9657	*599*	1-deoxy-D-xylulose-5-phosphate synthase [*Streptosporangium roseum* DSM 43021]	52	63	YP_003339378	1.08
RVR9665	*2535*	nonribosomal peptide synthetase [*Streptomyces paucisporeus*]	75	82	WP_073500751	0.45
RVR9671	*4908*	nonribosomal peptide synthetase [*Streptomyces paucisporeus*]	74	82	WP_073500750	0.46
RVR9672	*72*	mbtH-like protein [*Streptomyces paucisporeus*]	77	85	WP_073500749	0.23

Cluster 25. T1PKS

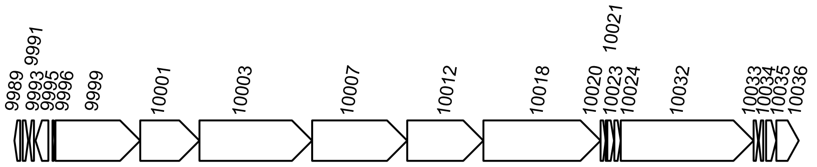

Gene	#AA	Putative function [blast hit species]	% identity	% positive	Accession number	Fold-expression
RVR9989	*324*	peptide ABC transporter permease [*Streptomyces yanglinensis*]	95	99	SEG91602	1.76
RVR9991	*314*	dehydrogenase [*Streptomyces yanglinensis*]	86	92	SEG91595	1.74
RVR9993	*251*	transcriptional regulator, GntR family [*Streptomyces yanglinensis*]	92	95	SEG91592	1.02
RVR9995	*718*	hypothetical protein [*Actinoplanes* sp. N902-109]	56	70	YP_007950495	1.35
RVR9996	*125*	hypothetical protein [*Actinoplanes* sp. N902-109]	63	80	YP_007950478	0.91
RVR9999	*4758*	polyketide synthase [*Streptomyces griseochromogenes*]	57	69	WP_067301771	1.38
RVR10001	*3320*	polyketide synthase [*Streptomyces griseochromogenes*]	57	69	WP_067301774	1.32
RVR10003	*6316*	polyketide synthase [*Actinoplanes* sp. N902-109]	55	67	WP_015620560	1.36
RVR10007	*5352*	polyketide synthase [*Actinoplanes* sp. N902-109]	59	71	WP_015620558	1.37
RVR10012	*4265*	polyketide synthase [*Amycolatopsis orientalis*]	55	66	ABM47020	1.21
RVR10018	*6582*	polyketide synthase [*Micromonospora echinospora*]	50	62	SCF29238	1.40
RVR10020	*296*	3-hydroxybutyryl-CoA dehydrogenase [*Actinoplanes* sp. N902-109]	80	88	YP_007950488	1.33
RVR10021	*95*	acyl carrier protein [*Actinoplanes* sp. N902-109]	70	78	YP_007950489	0.60
RVR10023	*372*	acyl-CoA dehydrogenase [*Actinoplanes* sp. N902-109]	68	80	YP_007950490	1.59
RVR10024	*370*	hypothetical protein [*Streptomyces griseochromogenes*]	71	82	WP_067301797	1.03
RVR10032	*7448*	polyketide synthase [*Pseudonocardia spinosispora*]	52	63	WP_051341806	1.39
RVR10033	*256*	4'-phosphopantetheinyl transferase [*Sinosporangium album*]	64	73	SDI30246	0.51
RVR10034	*252*	thioesterase [*Actinoplanes* sp. N902-109]	59	74	YP_007950477	0.80
RVR10035	*578*	ABC transporter [*Actinoplanes* sp. N902-109]	55	74	YP_007950501	1.75
RVR10037	*1265*	ABC transporter [*Actinoplanes* sp. N902-109]	61	74	YP_007950502	1.57

Cluster 26. T1PKS/NRPS

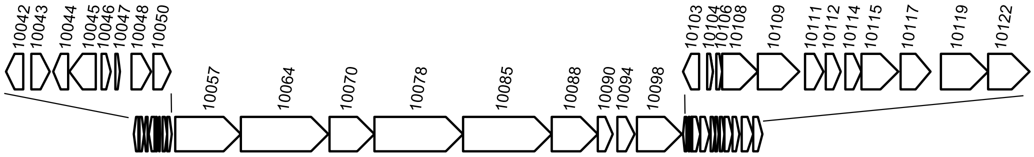

Gene	#AA	Putative function [blast hit species]	% identity	% positive	Accession number	Fold- expression
RVR10042	*294*	hypothetical protein [*Streptomyces rapamycinicus* NRRL 5491]	46	62	YP_008794661	0.86
RVR10043	*304*	transcriptional regulator [*Streptomyces viridochromogenes*]	47	58	WP_004004245	2.36
RVR10044	*251*	short-chain dehydrogenase [*Streptomyces tsukubaensis*]	77	85	WP_006347111	1.19
RVR10045	*447*	oxidoreductase [*Streptomyces afghaniensis*]	75	83	WP_020273778	1.28
RVR10046	*159*	cold-shock DNA-binding protein [*Streptomyces* sp. SirexAA-E]	60	71	YP_004801335	1.08
RVR10047	*76*	hypothetical protein [*Streptomyces* sp. NRRL B-1347]	70	81	WP_030679125	0.00
RVR10048	*322*	transcriptional regulator [*Streptomyces cellostaticus*]	85	93	WP_079058131	0.82
RVR10050	*304*	phosphoesterase [*Thermomonospora curvata* DSM 43183]	72	80	YP_003298132	1.59
RVR10057	*4862*	polyketide synthase [*Streptomyces griseochromogenes*]	60	71	WP_067301771	1.52
RVR10064	*6482*	polyketide synthase [*Streptacidiphilus carbonis*]	56	67	WP_042397185	1.36
RVR10070	*3308*	polyketide synthase [*Streptacidiphilus carbonis*]	59	69	WP_063770724	1.60
RVR10078	*6563*	polyketide synthase [*Pseudonocardia spinosispora*]	59	69	WP_037075676	1.37
RVR10085	*6529*	polyketide synthase [*Pseudonocardia spinosispora*]	59	69	WP_037075676	1.20
RVR10088	*3385*	polyketide synthase [*Streptomyces griseochromogenes*]	57	68	ANP49917	1.37
RVR10090	*1104*	nonribosomal peptide synthetase [*Streptomyces griseochromogenes*]	50	62	WP_079058161	1.19
RVR10094	*1279*	polyketide synthase [*Actinokineospora terrae*]	43	51	SER43109	0.85
RVR10098/PKS	*3311*	polyketide synthase [*Micromonospora pallida*]	53	63	SCL31511	1.44
RVR10103	*268*	thioesterase [*Streptomyces cellostaticus*]	69	78	WP_067010221	1.18
RVR10104	*112*	hypothetical protein [*Streptomyces cellostaticus*]	67	79	WP_079058130	1.00
RVR10106	*98*	hypothetical protein [*Streptomyces cellostaticus*]	70	87	WP_067010769	0.93
RVR10108	*588*	ABC transporter ATP-binding protein [*Actinoplanes* sp. N902-109]	61	75	YP_007950501	1.50
RVR10109	*698*	ABC transporter ATP-binding protein [*Actinoplanes* sp. N902-109]	61	75	YP_007950502	1.34
RVR10111	*320*	ABC transporter ATP-binding protein [*Streptomyces yanglinensis*]	86	91	SEG48332	1.20
RVR10112	*253*	integral membrane protein [*Streptomyces yeochonensis*]	72	84	WP_063836070	1.25
RVR10114	*267*	hypothetical protein [*Actinophytocola xinjiangensis*]	51	62	WP_075136491	1.96
RVR10115	*615*	AMP-binding enzyme [[*Actinophytocola xinjiangensis*]	62	70	WP_075136489	1.68
RVR10117	*515*	oxidoreductase [*Actinopolymorpha alba*]	53	69	WP_020578974	0.97
RVR10119	*786*	hypothetical protein [*Streptomyces yanglinensis*]	85	92	SEG55505	1.40
RVR10122	*690*	hypothetical protein [*Streptomyces* sp. HGB0020]	73	82	WP_016432827	1.39

Cluster 27. T1PKS/NRPS

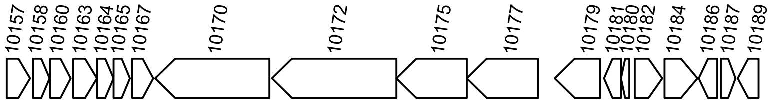

Gene	#AA	Putative function [blast hit species]	% identity	% positive	Accession number	Fold-expression
RVR10157	*385*	iron ABC transporter [*Streptomyces mangrovisoli*]	86	92	KKE95421	1.08
RVR10158	*271*	iron transporter ATP-binding protein [*Streptomyces mangrovisoli*]	84	91	WP_046585275	1.32
RVR10160	*334*	ABC transporter substrate-binding protein [*Streptomyces mangrovisoli*]	77	88	WP_052743021	1.34
RVR10163	*387*	ABC transporter ATP-binding protein [*Streptomyces alni*]	86	89	SFE52594	1.02
RVR10164	*271*	ABC transporter membrane protein [*Streptomyces alni*]	81	87	SFE52629	0.89
RVR10165	*270*	ABC transporter membrane protein [*Streptomyces alni*]	86	90	SFE52654	1.07
RVR10167	*343*	iron uptake ABC transporter substrate-binding protein [*Streptomyces alni*]	71	80	SFE52682	0.96
RVR10170	*1868*	nonribosomal peptide synthetase [*Streptomyces alni*]	70	76	SFE52711	0.87
RVR10172	*2035*	polyketide synthase [*Streptomyces alni*]	74	82	SFE52739	0.98
RVR10175	*1152*	nonribosomal peptide synthetase [*Streptomyces alni*]	75	80	SFE52769	0.88
RVR10177	*1163*	nonribosomal peptide synthetase [*Streptomyces alni*]	77	83	SFE52794	0.98
RVR10179	*740*	polyketide synthase [*Streptomyces alni*]	73	80	SFE52835	1.10
RVR10181	*274*	thioesterase [*Streptomyces alni*]	69	76	SFE52862	1.07
RVR10180	*137*	hypothetical protein [*Streptomyces alni*]	66	84	SFE52889	0.94
RVR10182	*449*	chorismate binding enzyme [*Streptomyces alni*]	81	85	SFE52918	0.96
RVR10184	*538*	AMP binding enzyme [*Streptomyces alni*]	80	87	SFE52947	0.96
RVR10186	*303*	siderophore-interacting protein [*Streptomyces alni*]	75	84	SFE52973	0.90
RVR10187	*241*	tetR family transcriptional regulator [*Streptomyces alni*]	72	81	SFE52996	1.07
RVR10189	*338*	hypothetical protein [*Streptomyces alni*]	75	82	SFE53018	0.87

Table S2. MALDI-TOF/MS analysis of the BR-1-binding protein RevU

Observed	Mr (expt)	Mr (calc)	ppm	Start	End	Peptide
885.4006	884.3933	884.4716	−88.5	440	448	R.AGDLPGSLR.L
1059.5258	1058.5185	1058.5972	−74.32	484	492	R.LTLELDVTR.L
1070.5584	1069.5512	1069.6318	−75.35	430	439	R.IMLSLVAPAR.A
1075.461	1074.4537	1074.5305	−71.47	622	632	R.SAAESAEAALR.S
1088.5444	1087.5371	1087.6237	−79.6	37	46	K.TTLLEAVVSR.A
1108.5433	1107.536	1107.6288	−83.76	745	754	R.LADEHLALVK.R
1120.4404	1119.4332	1119.5131	−71.42	580	589	R.LESAAAWCGR.L
1139.5312	1138.524	1138.5923	−60.07	680	688	R.FGLHYLYAR.G
1158.545	1157.5377	1157.6193	−70.49	727	736	R.TGAVEVWLQR.G
1165.5159	1164.5087	1164.5597	−43.86	189	199	R.LGAFDEVGMAR.L
1270.5179	1269.5106	1269.6201	−86.21	541	552	R.GPDDVLVAEAER.V
1293.5536	1292.5463	1292.6031	−43.89	324	335	R.SAVLDNMSAVDR.T
1351.7027	1350.6954	1350.8023	−79.15	528	539	R.LSAIHTLWAVLK.R
1479.6928	1478.6855	1478.7803	−64.09	795	806	R.YELALTLVELCR.T
1501.7028	1500.6956	1500.826	−86.91	275	290	R.GLAITNGAGSSTLLAR.L
1524.6709	1523.6636	1523.7634	−65.46	598	610	R.HAPTWHALFTSTR.A
1629.7676	1628.7603	1628.8886	−78.75	891	904	K.LFVTVSTVEQHLTR.A
1754.6961	1753.6888	1753.8424	−87.55	712	726	K.AQEWDLDSPALAPWR.T
1953.8313	1952.824	1953.0055	−92.91	855	873	R.SLPPSDDVVAGVLSEAELR.V
2289.9014	2288.8941	2289.0947	−87.63	461	483	R.VTDAVELMGPLFAGGADGTPAER.L
2333.9552	2332.9479	2333.1395	−82.14	633	654	R.SMSVEAWGVEIGMPLATAIEAR.T
2364.8648	2363.8575	2364.0257	−71.11	388	407	R.VSLAEQCLQLADNCCQDQGR.S
2405.9135	2404.9062	2405.0998	−80.48	691	711	R.HYLATGYYDAALADFIQCGEK.A
2582.0578	2581.0505	2581.2595	−80.95	655	679	R.TAMGDHAASAELVNSPVPPGLFQTR.F
2792.2481	2791.2408	2791.4446	−-73.01	493	516	R.LWLAFTYPGVHQQLVGDLPPEQQR.Q
2898.307	2897.2997	2897.5134	−73.76	553	579	R.VLQHLLAEDDTISSLNSAIAALVYADR.L
3448.6097	3447.6024	3447.7133	−32.16	348	380	R.NDGAPVIVVAQHLIAAGAVEDDWAPQFLMDAAK.Q

Amino acid sequences matching with RevU sequence are highlighted.

MLERDQCLDQLTELLDTCAEGNGATGVISGAVGFGK**TTLLEAVVSR**AAARGYMVLGAVGSKVESAIPYAVVEQLFQSAELAGA
ETDLHVPLQRAKEEWSHLGMEAGAAQAMQAYHALLVELSAHQPVVLCVDDIQHVDSESLACLLYLIRRCRRNPVTILLTLGPS
GAAPCEGVLAELACRPGVAHVR**LGAFDEVGMAR**LIADRFGPAAAEQYAPGFYAMSGGNPLLAQALVSDHTARPAHPETGYHPV
AADLFREASVACTRRSGLNGLRVAR**GLAITNGAGSSTLLAR**LVGLEKRDVDAAMKILTESRLIEGLRFRHPAIR**SAVLDNMSA**
VDRTRLHHRVAGLLR**NDGAPVIVVAQHLIAAGAVEDDWAPQFLMDAAK**QALREDR**VSLAEQCLQLADNC**CQDQGRSHAIKANL
ARIKWRDQPEAAAR**IMLSLVAPARAGDLPGSLR**LKVAHRLLVQGR**VTDAVELMGPLFAG**GADGTPAER**LTLELDVTRLWLAFT**
YPGVHQQLVGDLPPEQQRQVPSHQIGRPR**LSAIHTLWAVLK**R**GPDDVLVAEAERVLQHLLAEDDTISSLNSAIAALVYADRLE**
SAAAWCGRLQTAATER**HAPTWHALFTSTR**AMIALRRGSLR**SAAESAEAALRSMSVEAWGVEIGMPLATAIEARTAMGDHAASA**
ELVNSPVPPGLFQTRFGLHYLYARGR**HYLATGYYDAALAD**FIQCGEK**AQEWDLDSPALAPWRTGAVEVWLQR**GQRERAGR**LAD**
EHLALVKRGQRRTLGVALRVQAMTRPSSQQAHLLGTAVDMLQTAGDR**YELALTLVELCR**THQRQGAAAQARLLVRQAWRMASE
CGAEGLCSSLMPKPVAGVPVEQPR**SLPPSDDVVAGVLSE**AELRVCSLAARGHTNREISDK**LFVTVSTVEQHLTR**AYRKLNIRH
RRELPASLAS

Table S3. Bacterial strains, plasmids, and DNA used in this study

Strains, plasmids, and synthetic DNA templates	Relevant characteristics
**Strains**	
S. reveromyceticus SN-593[6,7]	Wild-type reveromycin A-producing strain
E. coli DH5α	Cloning host; F^- Φ80d*lacZ*ΔM15 Δ(*lacZYA-argF*)*U169 endA1 recA1 deoR hsdR17*($r_K^- m_K^+$) *phoA*, *supE44* λ^- thi-1 *gyrA96 relA1*
E. coli BL21 Star™(DE3)	Strain for recombinant protein expression; F^- *ompT hsdS*$_B$ ($r_B^- m_B^-$) *gal dcm rne131* (DE3)
E. coli BW 25113	*lacI*^{q} *rrnB*$_{T14}$ Δ*lacZ*$_{WJ16}$ *hsdR514* Δ*araBAD*$_{AH33}$ Δ*rhaBAD*$_{LD78}$ is derivative of the F^-, λ^-, *E.coli* K-12 strain BD792 (CGSC6159)
E. coli-conjugation	*E. coli* GM2929 *hsdS::*Tn10 carrying pUB307-*aph*::Tn*7*
Δ*revU* mutant	The *revU* gene disruptant from *S. reveromyceticus* SN-593
**Plasmids**	
pET28b(+)	T7 RNA polymerase-dependent recombinant protein expression vector (Novagen)
pET28b(+)-*revU*	The *revU* fragment (2772 bp) from *S. reveromyceticus* SN-593 was inserted into the NdeI and XhoI sites of pET28b(+).
pColdI-*revUsyn*	The codon-optimized *revU* gene fragment (2772 bp) for *E. coli* was inserted to the NdeI and HindIII sites of the pColdI vector (TaKaRa).
pCC1FOS™	The optimum vector for constructing a fosmid library (EPICENTRE® Biotechnology)
pKU250[8]	*E. coli – Streptomyces* conjugation vector
pIM[9]	The DNA region between the BamHI and KpnI sites was removed from pKU250.
pIM-*revU*	The vector has a 7784-bp insert DNA containing the *revU* gene in the middle.
pIM-Δ*revU*	The *revU* gene-disruption plasmid. The *revU* gene in pIM-*revU* was replaced by the *aphII* gene from the pKD13 plasmid.
pTYM-P$_{aph}$	The *aph* promoter fragment (327 bp) was inserted into the EcoRI and BamHI sites of pTYM19[10].
pTYM-P$_{aph}$-*revU*	The *revU* fragment (2772 bp) from *S. reveromyceticus* SN-593 was inserted into the BamHI and HindIII sites of pTYM-P$_{aph}$.
pKD46	Red recombinase expression plasmid[11]
pKD13	Template plasmid encoding the FRT-flanked kanamycin-resistant gene[11]
pWK	The β lactamase gene in the pWHM3 vector[12] was replaced with the *aphII* gene by λRed recombination.
pWK-P$_{tipA}$	The *tipA* promoter (P$_{tipA}$) was inserted into the EcoRI and BamHI sites of pWK
pWK-P$_{tipA}$-*revU*	The *revU* gene with a His-tag-coding sequence was amplified from pET28b(+)-*revU* and ligated into the BamHI and HindIII sites of pWK-P$_{tipA}$

Synthetic *revU* gene sequence

```
ATGCTGGAACGTGATCAGTGTCTGGATCAGCTGACCGAACTGCTGGATACCTGTGCCGAAGGTAATGGTGCAACCGGTGTTATTAGCGGTG
CAGTTGGTTTTGGTAAAACCACCCTGCTGGAAGCAGTTGTTAGCCGTGCAGCAGCACGTGGTTATATGGTTCTGGGTGCCGTTGGTAGCAA
AGTTGAAAGCGCAATTCCGTATGCAGTTGTTGAGCAGCTGTTTCAGAGCGCAGAACTGGCAGGCGCAGAAACCGATCTGCATGTTCCTCTG
CAGCGTGCAAAAGAAGAATGGTCACATCTGGGTATGGAAGCCGGTGCAGCACAGGCAATGCAGGCATATCATGCACTGCTGGTTGAACTGA
GCGCACATCAGCCGGTTGTTCTGTGTGTTGATGATATTCAGCATGTTGATAGCGAAAGCCTGGCATGTCTGCTGTATCTGATTCGTCGTTG
TCGTCGTAATCCGGTTACCATTCTGCTGACCCTGGGTCCGAGCGGTGCCGCACCGTGTGAAGGTGTTCTGGCCGAACTGGCCTGTCGTCCG
GGTGTTGCACATGTTCGTCTGGGTGCATTTGATGAAGTTGGTATGGCACGTCTGATTGCAGATCGTTTTGGTCCGGCAGCAGCAGAACAGT
ATGCACCGGGTTTTTATGCAATGAGCGGTGGTAATCCGCTGCTGGCACAGGCACTGGTTAGCGATCATACCGCACGTCCGGCACATCCGGA
AACCGGTTATCATCCGGTTGCAGCAGACCTGTTTCGTGAAGCAAGCGTTGCATGTACCCGTCGTAGCGGTCTGAATGGTCTGCGTGTTGCA
CGTGGTCTGGCAATTACCAATGGTGCAGGTAGCAGCACACTGCTGGCTCGTCTGGTTGGTCTGGAAAAACGTGATGTTGATGCAGCAATGA
AAATTCTGACCGAAAGTCGCCTGATTGAAGGTCTGCGCTTTCGTCATCCGGCAATTCGTAGCGCAGTTCTGGATAATATGAGCGCAGTTGA
TCGTACCCGTCTGCATCATCGTGTTGCTGGCCTGCTGCGTAATGATGGCGCACCGGTTATTGTTGTTGCACAGCATCTGATTGCTGCCGGT
GCGGTTGAAGATGATTGGGCACCGCAGTTTCTGATGGATGCAGCCAAACAGGCCCTGCGTGAAGATCGTGTTAGCCTGGCGGAACAGTGTC
TGCAGCTGGCAGATAATTGTTGTCAGGATCAGGGTCGTAGCCATGCAATTAAAGCAAATCTGGCACGTATTAAATGGCGTGATCAGCCGGA
AGCAGCAGCCCGTATTATGCTGAGCCTGGTTGCACCGGCACGTGCAGGCGATCTGCCTGGTAGCCTGCGTCTGAAAGTTGCACATCGTCTG
CTGGTGCAGGGTCGTGTTACCGATGCAGTGGAACTGATGGGTCCGCTGTTTGCCGGTGGTGCAGATGGTACACCGGCAGAACGTCTGACAC
TGGAACTGGATGTGACCCGTCTGTGGCTGGCATTTACCTATCCGGGTGTGCATCAGCAGCTGGTTGGTGATCTGCCTCCTGAACAGCAGCG
TCAGGTTCCGAGCCATCAGATTGGTCGTCCGCGTCTGAGCGCCATTCATACCCTGTGGGCAGTTCTGAAACGTGGTCCGGATGATGTTCTG
GTTGCCGAAGCAGAACGTGTTCTGCAGCATCTGCTGGCAGAAGATGATACCATTAGCAGCCTGAATAGCGCCATTGCAGCCCTGGTTTATG
CCGATCGTCTGGAATCAGCAGCAGCATGGTGTGGTCGCCTGCAGACCGCAGCAACCGAACGTCATGCACCGACCTGGCATGCACTGTTTAC
CAGCACCCGTGCAATGATTGCCCTGCGTCGTGGTAGTCTGCGTAGTGCAGCAGAAAGTGCAGAAGCAGCACTGCGTAGCATGAGCGTTGAA
GCATGGGGTGTTGAAATTGGTATGCCGCTGGCAACCGCAATTGAAGCACGTACCGCAATGGGTGATCATGCAGCAAGTGCGGAACTGGTTA
ATAGTCCGGTTCCGCCTGGTCTGTTCCAGACCCGCTTTGGTCTGCATTATCTGTATGCCCGTGGTCGTCATTATCTGGCCACCGGCTATTA
TGATGCCGCACTGGCAGATTTTATTCAGTGTGGTGAAAAAGCACAAGAGTGGGATCTGGATAGTCCGGCACTGGCACCGTGGCGTACCGGT
GCCGTGGAAGTGTGGCTGCAACGTGGTCAGCGTGAACGTGCAGGTCGTCTGGCAGATGAACATCTGGCCCTGGTTAAACGCGGACAGCGTC
GTACCCTGGGTGTTGCGCTGCGTGTGCAGGCAATGACCCGTCCGAGCAGCCAGCAGGCACATCTGCTGGGTACAGCAGTTGATATGCTGCA
AACAGCCGGTGATCGTTATGAACTGGCACTGACTCTGGTGGAACTGTGTCGTACCCATCAGCGCCAGGGTGCAGCTGCCCAAGCACGCCTG
CTGGTTCGTCAGGCATGGCGTATGGCAAGCGAATGTGGTGCGGAAGGTCTGTGTAGCAGTCTGATGCCGAAACCGGTGGCAGGCGTTCCGG
TTGAACAGCCACGTAGCCTGCCTCCGAGTGATGATGTTGTTGCCGGTGTTCTGAGCGAAGCCGAACTGCGTGTTTGTAGCCTGGCAGCGCG
TGGTCATACCAATCGTGAAATCAGCGATAAACTGTTTGTTACCGTTAGCACCGTTGAACAACATCTGACCCGTGCATATCGTAAACTGAAT
ATTCGTCATCGTCGTGAACTGCCTGCAAGCCTGGCCAGCTGA
```

Table S4. Sequences of primers used for qPCR

Gene	Forward primer (5' – 3')	Reverse primer (5' – 3')
revA	TTGTATGAAGAGTCGCCGGT	CATCCACCAGATCGAAAACC
revB	ACAAACCGGTGTTCGTCTTC	GAGCAGGTCCTCGTCGTC
revC	CGTGGTATTTGCACGAGTTG	GACAACTCACCCGTCAGACC
revD	GAGGATCGGGTTCTTGATGA	ATTCCGATACCAATACCCCC
revE	TCCTACTGCGACACCTTCGT	GAAGAAGTTCGAGCCGAGTC
revF	TCAACAGCTACGCCAACGA	AGATCGACTTCAGGACCTGGTA
revG	ACGACTCGACGGTAAGGTG	TCGGAGCCGAGGGAGTAG
revH	GCCGCGATCCGGTGGAAGAA	CCGGCGCTGATCGCCTTGAA
revI	GGAGCTGGTCGGCTACATC	TAGACGAAGCTGGGGATCTG
revJ	CCGGGTCTACGTCGTCAA	GAGCCCAGTTCGGAGATGTA
revK	AACCAGGTCGACAAGGTGAG	AGCTGTTCGTCGTTCTCGAT
revL	GAGTACACCGACCACGTCCT	GTTGAGGGTCTCCTTGAACG
revM	GAGACGCTGCTGGTCCAC	AGTCCTCGGTGGTGTAGTCG
revN	CAGCTGCACGAGCTGCCGAT	GCCGCCGTGGAAGAAGAGCA
revO	TTCGGCGCCATCGGCTACAC	GCCCAGCACCAGCAGCAGTT
revP	GAGGCCCTCCAGGAACTG	ATTGAGGGCGGAGTAGGTGT
revQ	CCAGGAGGTGCTAACGTACA	GAAGTCCGTGGCGTCGTG
revR	ATGTCCCACCTGGAGCAGT	AGGTCGATGATGTCGGTCA
revS	AAGACGATCCTCACCACGAC	GTGGAGCCCGAGGTGTACT
revT	TTCATCCACAGGTAGCGGTT	TCTTCGACTACATCGGCAAG
revU	CACCGACGCGGTCGAACTGA	GGTAGGTGAAGGCCAGCCACA
hrdB	CTTCACTCCGTCCTGGACAC	GTAGTCGCGCAACACCTGAG

References

1 Panthee, S., Takahashi, S., Hayashi, T., Shimizu, T. & Osada, H. β-carboline biomediators induce reveromycin production in *Streptomyces* sp. SN-593. *Sci. Rep.* **9**, 5802 (2019).

2 Takahashi, S. *et al.* Biochemical characterization of a novel indole prenyltransferase from Streptomyces sp. SN-593. *Journal of bacteriology* **192**, 2839–2851 (2010).

3 Takahashi, S. *et al.* Reveromycin A biosynthesis uses RevG and RevJ for stereospecific spiroacetal formation. *Nat. Chem. Biol.* **7**, 461–468 (2011).

4 Takahashi, S. *et al.* Structure-function analyses of cytochrome P450revI involved in reveromycin A biosynthesis and evaluation of the biological activity of its substrate, reveromycin T. *The Journal of biological chemistry* **289**, 32446–32458 (2014).

5 Miyazawa, T. *et al.* Identification of middle chain fatty acyl-CoA ligase responsible for the biosynthesis of 2-alkylmalonyl-CoAs for polyketide extender unit. *J. Biol. Chem.* **290**, 26994–27011 (2015).

6 Osada, H., Koshino, H., Isono, K., Takahashi, H. & Kawanishi, G. Reveromycin A, a new antibiotic which inhibits the mitogenic activity of epidermal growth factor. *J. Antibiot.* **44**, 259–261 (1991).

7 Koshino, H., Takahashi, H., Osada, H. & Isono, K. Reveromycins, new inhibitors of eukaryotic cell growth. III. Structures of reveromycins A, B, C and D. *J. Antibiot.* **45**, 1420–1427 (1992).

8 Komatsu, M., Uchiyama, T., Omura, S., Cane, D. E. & Ikeda, H. Genome-minimized *Streptomyces* host for the heterologous expression of secondary metabolism. *Proc. Natl. Acad. Sci. USA.* **107**, 2646–2651 (2010).

9 Takahashi, S. *et al.* Biochemical characterization of a novel indole prenyltransferase from *Streptomyces* sp. SN-593. *J. Bacteriol.* **192**, 2839-2851 (2010).

10 Onaka, H., Taniguchi, S., Ikeda, H., Igarashi, Y. & Furumai, T. pTOYAMAcos, pTYM18, and pTYM19, actinomycete-*Escherichia coli* integrating vectors for heterologous gene expression. *J. Antibiot.* **56**, 950–956 (2003).

11 Datsenko, K. A. & Wanner, B. L. One-step inactivation of chromosomal genes in *Escherichia coli* K-12 using PCR products. *Proc. Natl. Acad. Sci. USA.* **97**, 6640–6645 (2000).

12 Vara, J., Lewandowska-Skarbek, M., Wang, Y. G., Donadio, S. & Hutchinson, C. R. in *Journal of bacteriology* Vol. 171 5872-5881 (1989).
